# Material Stock and Embodied Greenhouse Gas Emissions of Global and Urban Road Pavement

**DOI:** 10.1021/acs.est.2c05255

**Published:** 2022-12-01

**Authors:** Lola S. A. Rousseau, Bradley Kloostra, Hessam AzariJafari, Shoshanna Saxe, Jeremy Gregory, Edgar G. Hertwich

**Affiliations:** † Industrial Ecology Programme, Department of Energy and Process Engineering, NTNU − Norwegian University of Science and Technology, Høgskoleringen 5, 7034 Trondheim, Norway; ‡ Department of Civil & Mineral Engineering, University of Toronto, 35 St. George Street, Toronto, OntarioM5S 1A4, Canada; § School for Environment and Sustainability, University of Michigan, Dana Building, 440 Church Street, Ann Arbor, Michigan48109, United States; ∥ Civil & Environmental Engineering, Massachusetts Institute of Technology, 77 Massachusetts Avenue, Cambridge, Massachusetts02139, United States; ⊥ MIT Climate and Sustainability Consortium, Massachusetts Institute of Technology, 105 Broadway Street, Cambridge, Massachusetts02142, United States

**Keywords:** anthropogenic material stock, built environment, road, pavement, transport infrastructure, urban areas, resource use, bottom-up approach

## Abstract

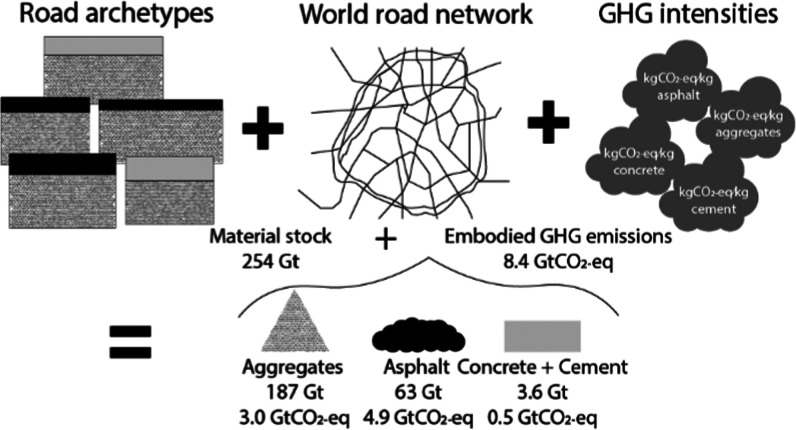

Roads play a key role in movements of goods and people but require large amounts of materials emitting greenhouse gases to be produced. This study assesses the global road material stock and the emissions associated with materials’ production. Our bottom-up approach combines georeferenced paved road segments with road length statistics and archetypical geometric characteristics of roads. We estimate road material stock to be of 254 Gt. If we were to build these roads anew, raw material production would emit 8.4 GtCO_2_-eq. Per capita stocks range from 0.2 t/cap in Chad to 283 t/cap in Iceland, with a median of 20.6 t/cap. If the average per capita stock in Africa was to reach the current European level, 166 Gt of road materials, equivalent to the road material stock in North America and in East and South Asia, would be consumed. At the urban scale, road material stock increases with the urban area, population density, and GDP per capita, emphasizing the need for containing urban expansion. Our study highlights the challenges in estimating road material stock and serves as a basis for further research into infrastructure resource management.

## Abstract 2

We estimate the global and urban road material stock. Per-capita stock varies significantly, highlighting a future potential growth in road material stock suggesting a necessity for high resolution assessment.

## 1 Introduction

Roads have direct and indirect contributions in fulfilling the Sustainable Development Goals (SDGs).^1^ Targets 3.6, 9.1, and 11.2 address road safety, accessibility to road networks in rural areas, and public transport to provide a safer and better connectivity to economic and social activities.^2,3^ Enhancing movements of people and goods contributes to fulfilling other SDGs by providing access to essential goods and services such as education and health centers.^3^

Increasing paved road surface area is expected as ∼14% of the global population does not have access to all season road infrastructure.^4^ However, the development of roads has environmental consequences. Roads contribute to the spread of impervious surfaces that prevent the infiltration of water into the soil,^5^ consume large amounts of resources for their construction and maintenance,^6−8^ contribute to habitat fragmentation and many other ecological impacts.^9^ Roads also have a long lifetime and may lock in patterns of land use, energy consumption, and greenhouse gas (GHG) emissions.^10−12^ In the literature on material stock in the built environment, Lanau et al.^13^ collected road focused studies at the urban, regional, and national scales but did not retrieve any study focusing specifically on roads at the global scale. In a literature review of Material Flow Analysis (MFA) of road networks by Ebrahimi et al.,^14^ 16 studies are collected and only one study is on a global scale but adopts a top-down approach by estimating the material stock in roads using bitumen production as a proxy.^15^ To the best of our knowledge, only the study from Virág et al.^16^ provides a bottom-up estimate of road material stock at the global scale. There is therefore room for further research on road material stock.

Countries are not homogeneous entities in terms of how population, economic activities, and transport infrastructure are geographically distributed. Roads’ geospatial distribution is required for a detailed estimation of their environmental impacts.^4,17^ Spatial patterns of road materials enrich our understanding of material stock and flows in the built environment and provide insights about future road material use for developing strategies towards sustainable use of resources.^18^ Moreover, population and economic activities are concentrated in urban areas requiring large stock and flows of materials.^13^ Drivers behind roads networks and their development have been explored through multivariable regression models of road length at the national level^19,20^ or power-law scaling relationships between road length/road surface and the city size (often described by its population).^21−24^ Understanding the relationship between material use and socio-economic characteristics of urban areas becomes crucial to develop strategies for reducing material use and greenhouse gas emissions in urban environments.

The aim of this paper is two-fold: (1) provide an estimate of the global road material stock (and the GHG emissions associated with the production of those materials), and (2) provide insights on the relationship between road material stock in urban areas and socio-economic characteristics. The next section describes the Materials and Methods we have used to fulfil these two objectives. Section 3 presents the results and the discussion.

## 2 Materials and Methods

**Figure 1:**
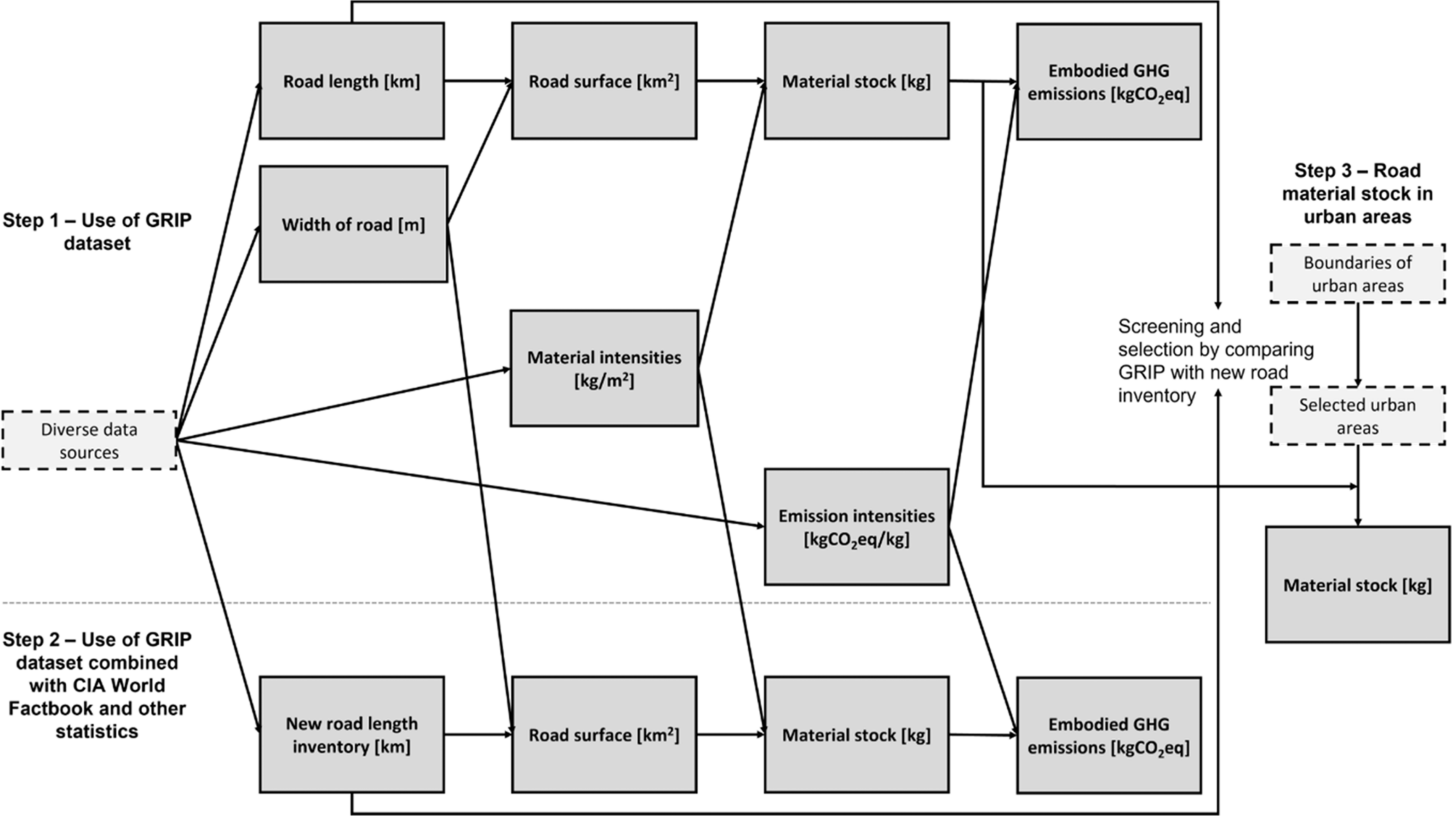
Step 1: Estimate and spatially distribute the material quantities and embodied GHG emissions of the global road network using GRIP dataset; Step 2: Combining GRIP dataset with additional road length statistics to provide a new road inventory; Step 3: Estimate road material stock in urban areas. A detailed version of this figure is available in the Supporting Information.

This paper is decomposed into three steps (Figure 1): (1) providing a first estimate of material stock and embodied GHG emissions using geographically referenced road networks, (2) generating a new road inventory combining the spatially distributed road networks with additional statistics, and (3) estimating the material stock in urban areas.

### 2.1 Data Collection and Road Archetypes

The length, width, and pavement profile (thicknesses of layers composing the pavement) of roads were combined (Figure 1 – Step 1) to estimate stocks of road materials (asphalt, granular, concrete, cement). Materials’ stocks were then multiplied with the GHG emissions intensities of raw material production to calculate embodied GHG emissions of pavement. The model is shortly described in this section, details are provided in the Supporting Information (Sections S1–S3).

For road length and spatial distribution, the Global Road Inventory Project (GRIP) dataset^20^ was used. Road segments in GRIP are in one of the seven regions: (1) North America, (2) Central and South America, (3) Africa, (4) Europe, (5) Middle East and Central Asia, (6) South and East Asia, and (7) Oceania. The GRIP dataset provides a harmonized classification of the road segments into five road types: (1) highways, (2) primary, (3) secondary, (4) tertiary, and (5) local roads. The initial intention was to limit the analysis to road segments classified as “paved”. However, this would lead to excluding all roads located in China (as they are listed as “unspecified surface” – Figure S3). We therefore included in our analysis “paved” roads from all countries as well as all “unspecified surface” roads in China.

Climate conditions are known to influence road design and construction standards.^25^ The GRIP dataset was combined with a climate zones raster file (Beck et al.^26^) to provide information on the climate conditions of the roads segments. The climate zones are then aggregated into four climate classes: (1) wet-freeze, (2) wet-non freeze, (3) dry-freeze, and (4) dry-non freeze (Table S1).

Roads have varying geometric characteristics due to differences in traffic volume and speed, and in construction norms across regions. The road segments were supplemented with archetypical attributes: width (in meters) and material intensities (kg/m^2^ of road surface) depending on their pavement surface (asphalt/concrete). These attributes represent in a simple model the construction of roads according to the country, the climate class, the road type, as well as the pavement type.

Width was calculated based on the number of travel lanes on a road segment, retrieved from OpenStreetMap (OSM) through the Python library Pyrosm^27^ (details in Section S2.1). For each country and road type, after considering that some roads are dual carriageways in OSM, the weighted average number of lanes from OSM is calculated and associated to each GRIP road segment (Table S3). A typical width of one lane is assumed to be ∼3.5 m (Table S5). However, lane width varies by road type and location. To capture this uncertainty and account for the possible presence of shoulders and auxiliary lanes not captured in OSM lane counts, the width of a road segment was calculated as being within a range of values (Table S4).

The material intensities (kg/m^2^) for the road segments were estimated through data collection of pavement cross section from design codes and scientific literature (Table S6), combined with densities of materials. Lower and upper values of material intensities were estimated to consider uncertainty. The data collection was limited to a few countries in each GRIP region due to resource constraints, and countries for which data were collected were used as proxies for other countries in the same region (e.g., South Africa was used as a proxy for countries located in Africa). Material intensities for asphalt pavement and concrete pavement were then combined into single material intensities by applying ratios of asphalt versus concrete pavements in each country (Table S7).

Asphalt GHG emissions were estimated by combining GHG emissions from crude oil extraction^28^ with crude oil trade and production^29,30^ as well as with other inputs (based on modified processes from Ecoinvent v3.6^31^) required in asphalt production (Section S3.1). Granular material GHG emissions intensities were assumed to be the same as for the aggregates used in the asphalt mixture (Section S3.1.2). GHG emissions intensities of concrete and cement were also based on modified processes from Ecoinvent v3.6^31^ (Tables S16 and S17). The intention was to get, to the furthest extent, region (or country) specific GHG factors. For a country, when its country specific GHG factor was not available, the region specific one (depending on the GRIP region the country belongs to) was used.

### 2.2 Combination of GRIP Dataset with Additional Statistics

The GRIP dataset has its limits due the limited availability of georeferenced road networks in some regions.^20^ Paved road length from CIA World Factbook^32^ (providing country level roadways length) and additional statistics^33−42^ is compared with the GRIP paved road length and total road length (including other types of road surfaces) to provide an estimate of the missing road length. Our assumptions are: (1) the paved road length collected from CIA World Factbook or other statistics, are only comprising roads and their length does not double count dual carriageways; (2) roads of higher-level classification are more probable to be paved. In the GRIP dataset, roads have been represented as single lines (including dual carriageways which are represented as two parallel lines in OSM). Therefore, the comparison is immediate. Three situations are then encountered:

- If the paved road length collected from CIA World Factbook or from other statistics is lower than GRIP paved road length, nothing is done.
- If the paved road length collected from CIA World Factbook or from other statistics is comprised between GRIP paved road length and GRIP total road length, we add non paved road length from total GRIP starting from highways up to local roads until the sum reaches the new paved road length.
- If the paved road length collected from CIA World Factbook or from other statistics is larger than total GRIP, then all segments from GRIP are considered and the rest of missing length is added as local roads (most of the roads missing are local roads as suggested by the authors from GRIP and this would provide a lower estimate of the material stock/GHG emissions).

The road lengths calculated by road type are then disaggregated by climate type using the initial climate shares for each road type from GRIP (or the global climate share of the country if a road type is not available in paved GRIP). Road archetypes are then applied to get the material stock/GHG emissions as presented in Figure 1 (Step 2).

**Figure 2:**
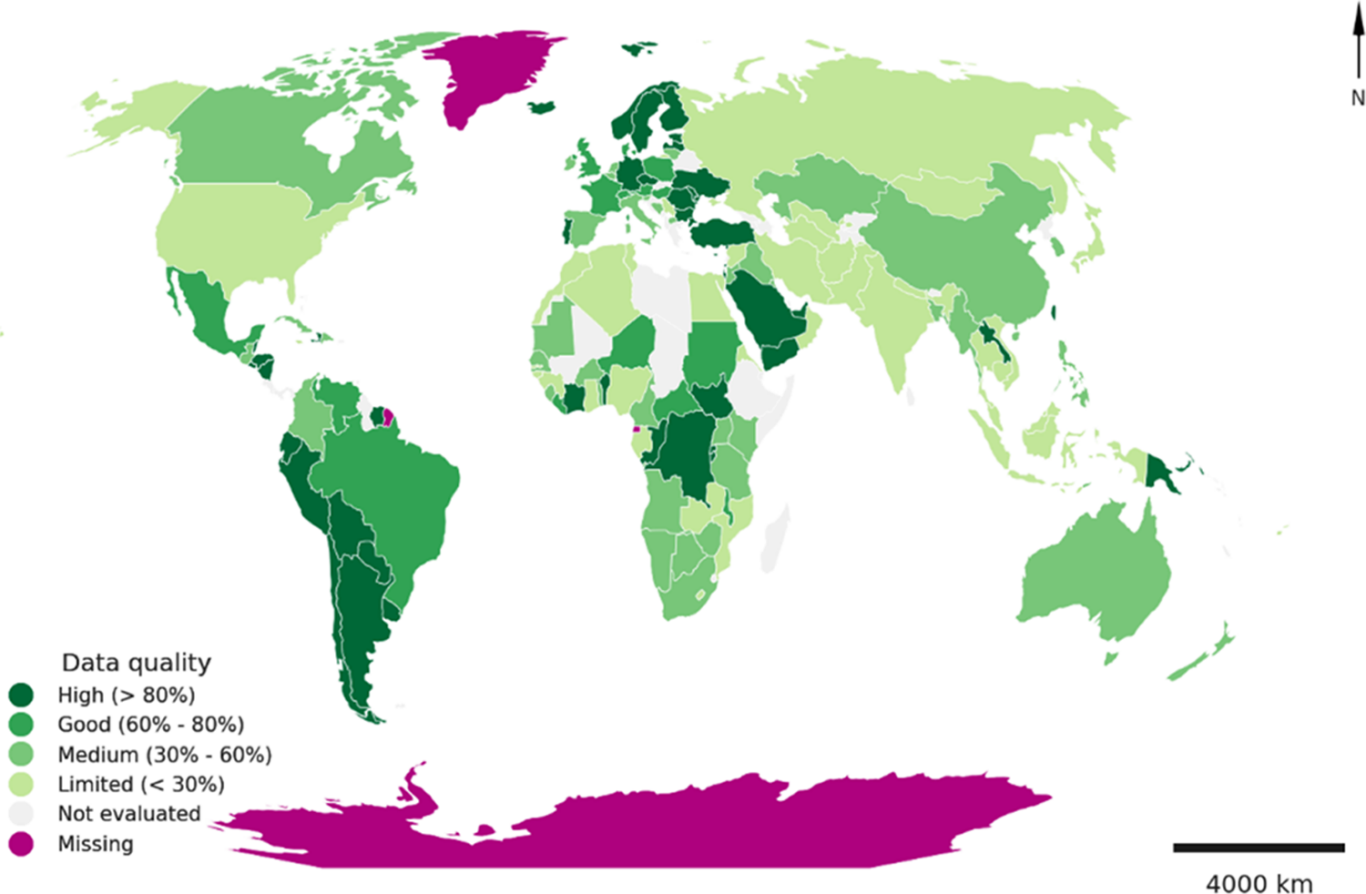
Data quality: How does paved GRIP dataset (and unspecified surface roads in China) compare to the newly generated paved road inventory. The category “Not evaluated” means that either there is no external paved road length value available to compare with the value from GRIP dataset, or there is no paved length reported by GRIP (but there is external paved road length reported).

Combining GRIP dataset with additional statistics leads to a new inventory of roads disaggregated by road type for each country. Based on this new road inventory, we classify countries based on their data quality (i.e., what percentage of road length is covered by GRIP) as shown on Figure 2. We find that in many countries, 20% or more of the road length is not covered by GRIP with the highest coverage (>80%) in Central and South American and European countries.

Nonetheless, some limitations have to be highlighted. The use of the CIA World Factbook^32^ is convenient as it is publicly available and reports the length of paved roads but the database lacks transparency (e.g., does not cite their data sources^43^), it reports road lengths of different years, and there might be inconsistencies in the way road length has been reported for different countries (countries might measure their road length differently^20^ and/or might have different definition of paved roads^19^). In addition, official statistics do not necessarily capture all roads, especially low-class roads,^44^ leading to underreporting of the road length. Therefore, using spatially distributed road segments provides more reliable length of paved roads (when the surface type is indicated). This also provides spatial information on road material distribution which can be used for further analysis as for evaluating road material stock in urban areas.

### 2.3 Roads in Urban Areas

Combining Geographic Information System data on roads with material stock provides insights on the use and environmental impacts of the existing infrastructure as well as the deployment of new infrastructure.^45^

The role of highways in suburbanization and urban areas expansion has been investigated. Baum-Snow^46^ demonstrates how the construction of highways led to depopulation of city centres in the United States between 1950 and 1990. Garcia-López et al.^47^ perform a similar study for Spanish cities and show the combined effect of new highways in depopulating city centres and attracting new population in the suburban areas. Roads influence how urban areas are developing and where population is living. Urban areas have high concentration of material stock.^13^ A better knowledge of material stock’s spatial distribution would inform urban planners and policy makers towards sustainable urban planning^48^ and highlight future opportunities for urban mining.^13,45^ An analysis of the relationship between urban areas socio-economic characteristics and their total road material stock would also provide insights on why urban areas have different road material stock.

Meijer et al.^20^ found that road length in a country increases with its land surface area, population density, Gross Domestic Product (GDP), and OECD membership. We want to evaluate if these results hold for road material stock at the urban level. Analyzing urban road material stock depends on having geospatial data for roads (to discern roads belonging to each urban area from all other roads), thus the GRIP dataset is used exclusively. The centroids of paved road segments from GRIP supplemented with their material stocks are spatially joined with polygons of urban areas^49^ to calculate road material stock for each urban area (the response variable of our analysis).

The explanatory variables tested are the urban surface areas, the population density, and the GDP per capita. These variables were already part of Meijer et al.’s^20^ analysis. These variables are not only selected for our regression analysis because they cover population and affluence characteristics,^20^ but because they are easily accessible for any urban area with global spatial distribution available^50,51^ (details in Section S6.1).

To provide a sound estimation of how the selected socio-economic variables are related to the road material stock, urban areas located in countries with data quality “good” and “high” are selected (as shown on Figure 2, “good” quality data refers to GRIP dataset covering 60–80% of the new road length inventory generated by combining GRIP with additional statistics, and “high” quality data refers to GRIP dataset covering more than 80% of this new inventory). To limit outliers, we filter the dataset to urban areas having a level of urbanization of at least 5000 inhabitants and 300 inhabitants per km^2^ (following Eurostat’s definition for urbanized areas^52^) resulting in a filtered dataset of 2204 urban areas distributed in five regions (Central and South America, Europe, Africa, Middle East and Central Asia, and South and East Asia). Two regions comprise the largest datasets (1287 urban areas in Europe and 731 in Central and South America) with the most country diversity (Figure S7).

Equation 1 relates urban road material stock to the characteristics described above, which is then log-transformed into eq 2 to linearize the relationship and minimize skewed distributions. By multiplying socio-economic variables and applying them exponents to get the road material stock, this equation can be considered as a derivative of the STIRPAT equation.^53^
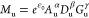
1
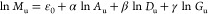
2

For each urban area u, *M*_u_ is the total stock of road materials (kg), *A*_u_ is the urban surface area (km^2^), *D*_u_ is the population density (cap./km^2^), and *G*_u_ is the GDP per capita (constant 2011 international US dollars/cap.). The parameters ε_0_, α, β, and γ are determined by the regression analysis.

Multivariable Ordinary Least-Squares (OLS) regression models are computed for Europe and Central and South America using the Python library statsmodels v0.12.2.^54^ To verify the validity of our regression models, the urban areas datasets are split into two subsets, a training set and a test set, using the Python library scikit-learn v0.24.2.^55^

## 3 Results and Discussion

The results are divided into (1) the material stock and embodied GHG emissions in the global road network, and (2) the urban area multivariable regression analysis.

### 3.1 Material Stock and Embodied GHGs in the Global Road Network

**Figure 3:**
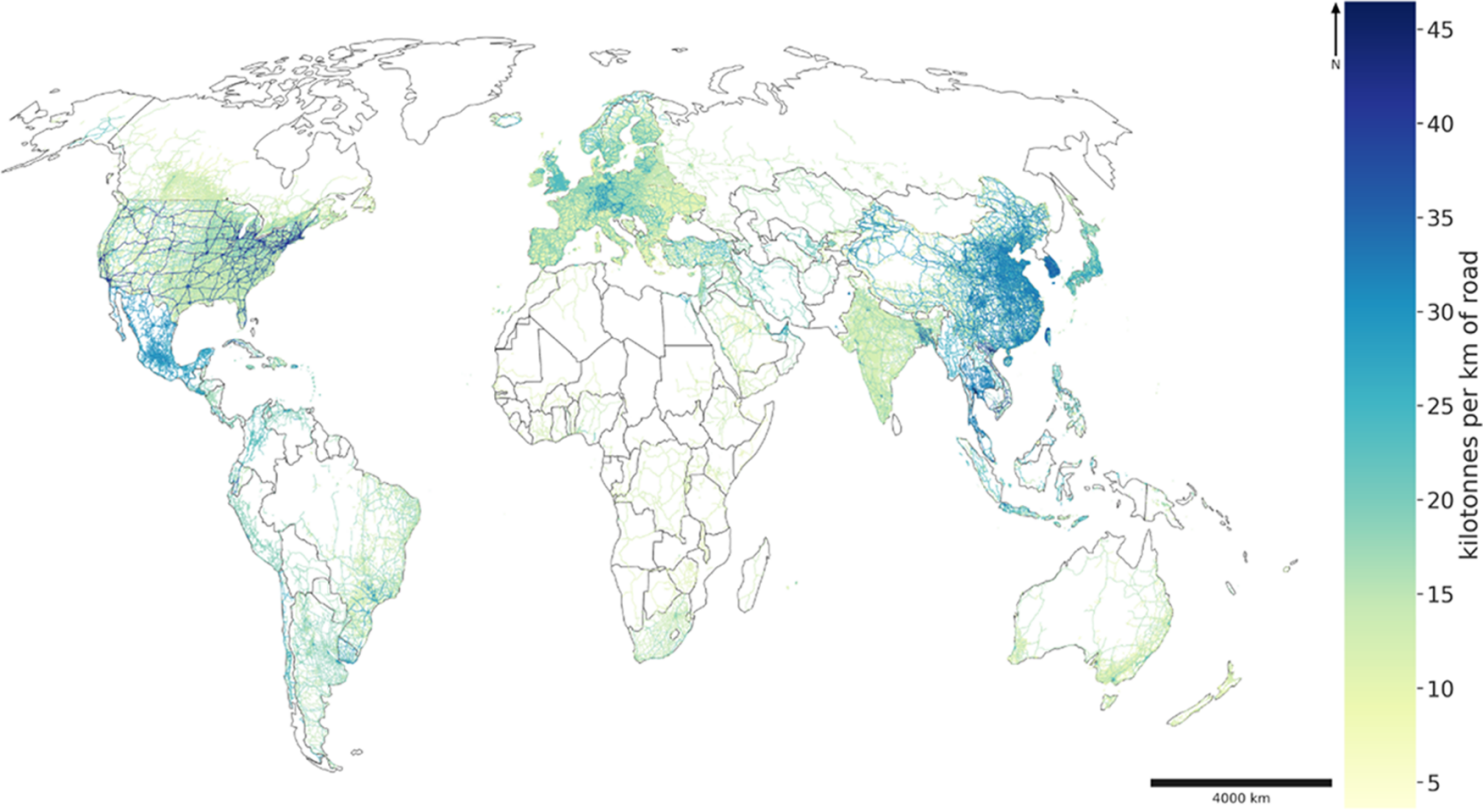
Map of the paved road network based on GRIP dataset – Material stock per km of road (kilotonnes per km of road). The GRIP dataset is based on OpenStreetMap version of 2015 supplemented with other data sources (ranging between years 1997 and 2015).

Figure 3 is a map of the paved road network using GRIP dataset. Colors indicate the material stock per km. Roads of highest material intensity are in North America and East Asia. Within each country, roads of a same type and same climate class have the same material composition owing to assumptions about national road profile built into the adopted archetypes.

**Figure 4:**
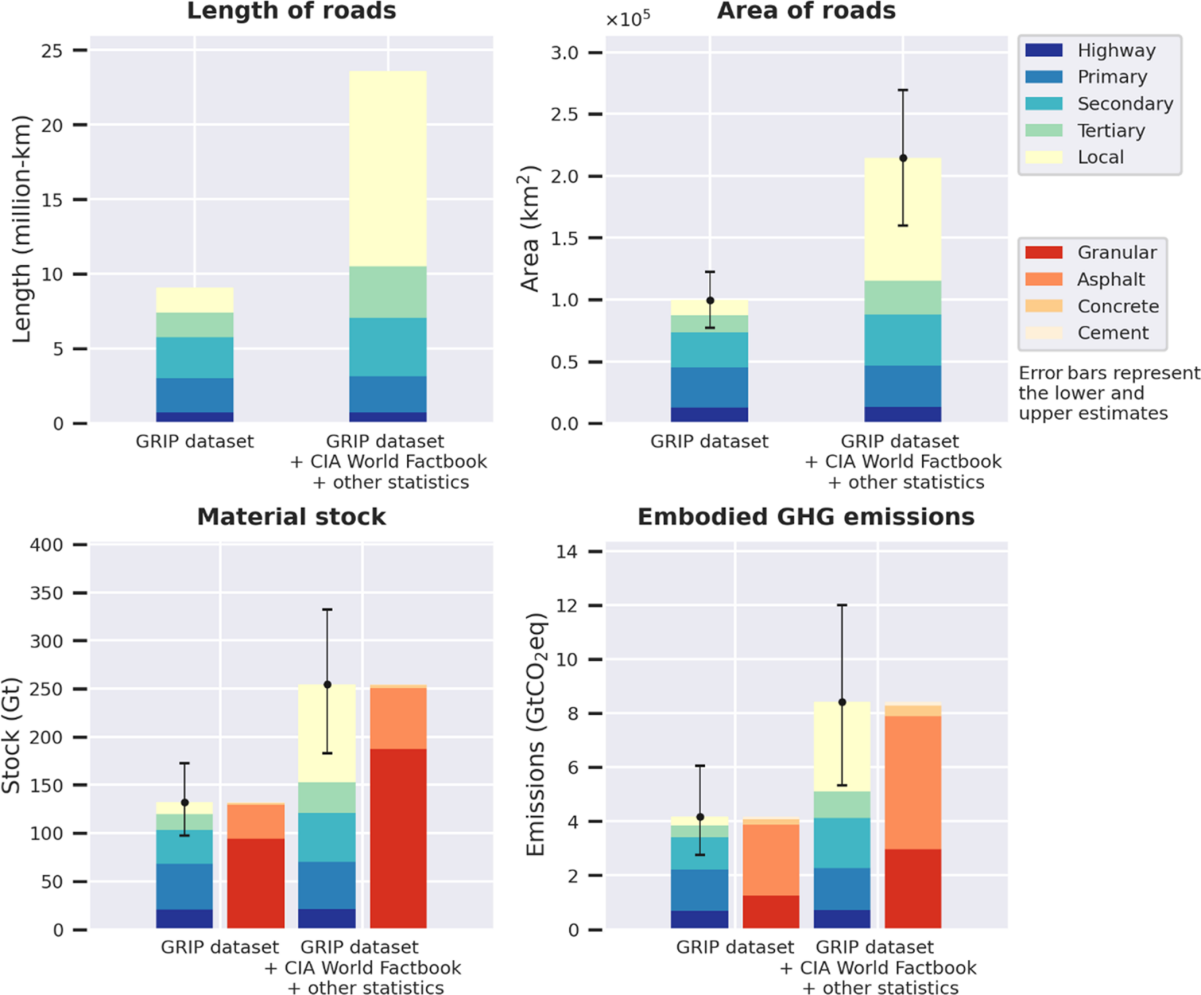
Comparison of paved road segments based on GRIP dataset and the combination with data from CIA World Factbook^32^ and other statistics.^33−42^ The two graphs at the bottom present the material stock and emissions disaggregated by road type and by material type (Gt is Gigatonnes with tonnes corresponding to metric ton (i.e.) 1000 kg).

Figure 4 compares GRIP dataset to its combination with paved road length retrieved from CIA World Factbook^32^ and other statistics.^33−40^ GRIP dataset covers only around 46% of paved road area, and 50% of materials and embodied GHG emissions from the current road network. Figure 4 shows that local roads are driving this difference. This can be explained by the re-estimation algorithm in which local roads were added to adjust the total road length if the global GRIP dataset (with non-paved surfaces) was not sufficient to reach the total paved road length reported by CIA World Factbook^32^ or other statistics.^33−40^Figure 4 also shows that granular is the material with the largest stock (granular: 187 Gt, asphalt: 63 Gt, concrete: 3.5 Gt, cement 0.1 Gt) but asphalt is the largest contributor to the embodied GHG emissions (asphalt: 4.9 GtCO_2_-eq, granular: 3.0 GtCO_2_-eq, concrete: 0.4 GtCO_2_-eq, cement 0.1 GtCO_2_-eq).

To place our results in perspective, Table 1 presents the population weighted-average material stock and GHG per capita for each GRIP region.

**Table 1:** Per Capita Population Weighted-Average Material Stock (Tonnes/Capita) and GHG (Tonnes CO_2_-eq/Capita)

GRIP region	population weighted-average material stock per capita (tonnes/capita)	GHG per capita (tonnes CO_2_-eq/capita)
North America	144	5.4
Central and South America	24	0.7
Africa	5	0.2
Europe	69	2.2
Middle East and Central Asia	37	1.9
South and East Asia	29	0.8
Oceania	143	5.6

More specifically, countries with the largest material stock per capita are found in Northern Europe. Iceland (283 t/cap), Lithuania (266 t/cap), and Finland (192 t/cap) are at the top. Countries with lowest material stock are Chad (0.2 t/cap), Solomon Island (0.5 t/cap), and Ethiopia (0.9 t/cap). The large variance in estimated per capita road stock among countries (Weighted Mean = 35 t/cap, Weighted Variance = 1190 t/cap) supports previous findings on inequalities in access to roads.^4,20^ About 166 Gt of additional material would be needed by 2050 to equip Africa’s projected population of nearly 2.5 billion^56^ with the same level of per capita paved road stock existing in Europe today (69 t/cap), an increase of nearly 2900% compared to the current stock, a similar amount of road material in North America and in East and South Asia. This highlights an important challenge for the 21st century, to find a way for countries that currently have disproportionate low access to infrastructure to gain access to infrastructure services without following the same destructive resource consumption patterns of 20th century development.

### 3.2 Multivariable Regression Analysis of Material Stock in Urban Areas

**Figure 5:**
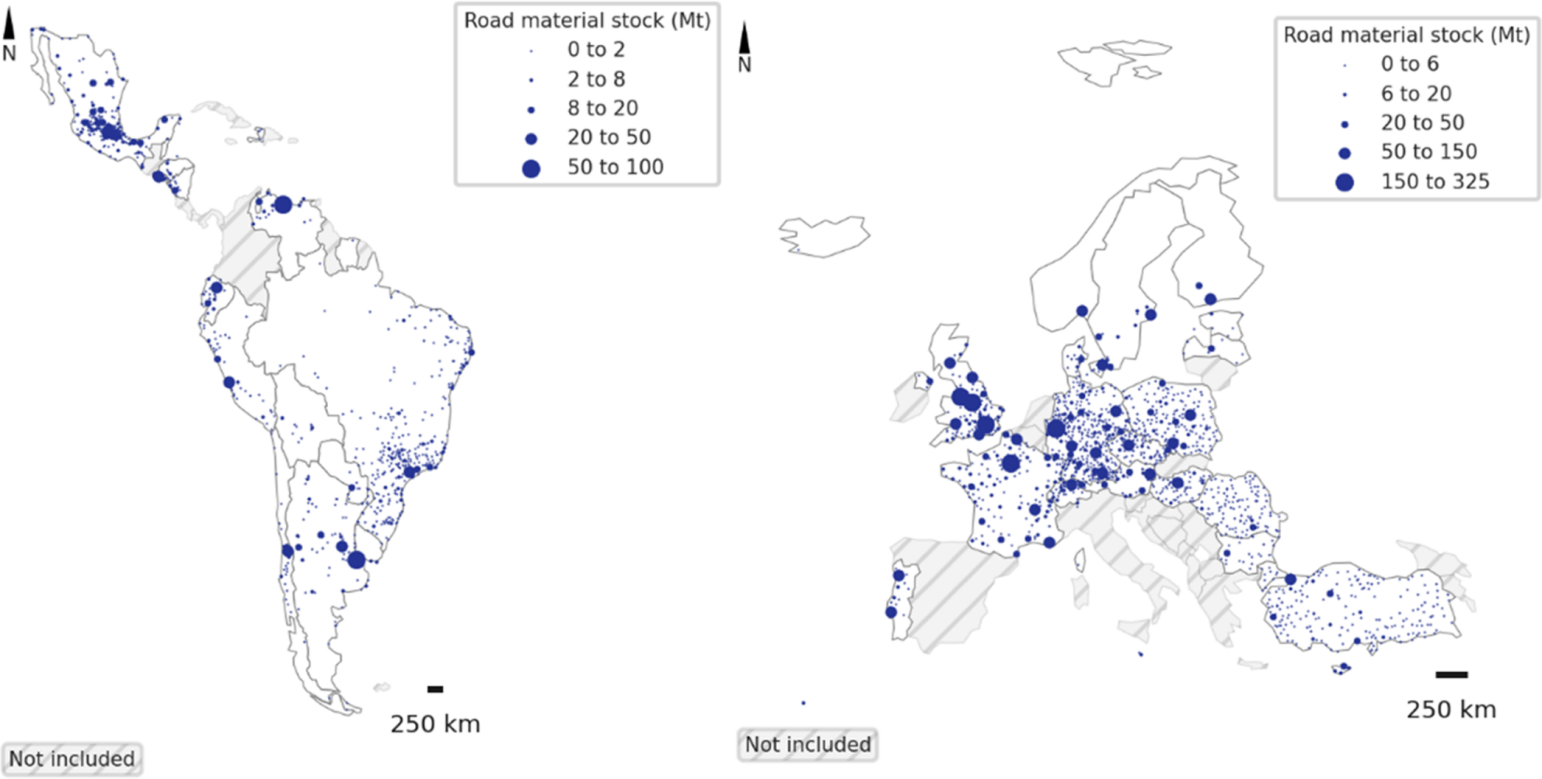
Urban areas included in the regression analysis (centroids of urban areas are shown on the map).

Having presented the results at the national scale, this section focuses on roads at the urban scale. The urban areas in Europe and Central and South America included in the regression analysis are shown on Figure 5.

The final regression model based on the selected urban areas is presented in Table 2.

**Table 2:** Results of the OLS Multivariable Regression Model Using the Python Library Stasmodels v0.12.2^54^

	Central and South America		Europe	
explanatory parameters	value	confidence interval	value	confidence interval
intercept, ε_0_	13.546***	[13.098–13.994]	4.766***	[4.034–5.498]
urban area, α	1.207***	[1.124–1.290]	1.061***	[1.033–1.089]
density, β	NA^a^		0.312***	[0.243–0.381]
GDP per capita, γ	NA^a^		0.938***	[0.880–0.996]
*R*-squared	0.53		0.86	

a NA indicates that density and GDP per capita are not statistically significant for Central and South America. ***p-value < 0.001.

Road material stock in European urban areas scales almost linearly with the urban area and the GDP per capita (Table 2). It also increases with the density, however, at a lower degree. Regarding Central and South America, only the urban area presents a superlinear relationship with urban material stock (α > 1). With the data available, it appears that density and GDP per capita do not explain the variation in road material stock for Central and South American urban areas. For both regions, ln(*A*_u_) is correlated with ln(*M*_u_) with Pearson correlation coefficients of 0.73 for Central and South America and 0.86 for Europe (Figure S8) revealing a close relationship between roads and size of urban areas. The socio-economic variables not being correlated as much as the urban area (Pearson correlation coefficients ranging between 0.09 and 0.36 for both variables in both regions) and not being statistically significant for Central and South America agrees with Meijer et al.^20^ who indicate that increasing socio-economic variables might lead to a more intensive use of the existing network in addition to new roads.

### 3.3 Discussion

We have estimated the global road material stock and its embodied GHG emissions: 254 Gt (lower estimate 183 Gt and upper estimate 332 Gt) and 8.4 GtCO_2_-eq (lower estimate 5.3 GtCO_2_-eq and upper estimate 12 GtCO_2_-eq) which is in between direct GHG emissions in the United States and China in 2019.^57^

We compare our results with literature. Virág et al.^16^ and Wiedenhofer et al.^58^ estimated the global road material stock to be of 294 Gt (including tunnels, bridges, and gravel roads). The comparison with our estimated stock is therefore not direct. High-class roads for which we obtained 121 Gt can be compared with Virág et al.’s estimation^16^ of 133 Gt. For low-class roads, we obtained 133 Gt while Virág et al.^16^ estimated 146 Gt comprising gravel roads. Despite a different scope, the estimations are still within the same order of magnitude. National road material stock per capita from our study (ranging between 0.2 and 283 t/cap.) are compared with material stock per capita from prior studies i.e., studies modeling material stocks and flows of road infrastructure or modeling material stock in the built environment including roads at the national or city level (Section S7.1). Ebrahimi et al.^14^ highlight three reasons to explain differences between studies about road material stocks: (1) road infrastructure coverage, (2) materials included, and (3) material intensities. The scope to estimate material stock of the built environment might differ greatly depending on data availabilities and purpose of the study. Different road types can be included (e.g., for Norway we included European, national, county, and municipal paved roads while Ebrahimi et al.^14^ did not include municipal roads), different facilities can also be included (only the pavement is included in our work while Haberl et al.^44^ included all road elements e.g., parking lots, footpaths, pedestrian streets, etc.), and material intensities which, when using an archetype modeling approach, are based on heterogenous data collection and subjective choices.

Archetypes are useful to represent in a simple manner the typical structure of roads. This approach receives criticism because of its oversimplification and time consumption if one wants to define detailed archetypes.^14^ An alternative to archetypes is to collect detailed consumption data about road construction, collect empirical data from municipal or governmental agencies,^59^ or use of machine learning^14^ but this requires large datasets. Therefore, the use of archetypes enables a first estimation of global material stock. To define our archetypes, data have been collected specifically for this project and from secondary sources of data. Our archetypes are differentiated by country, road type (for number of lanes, lane width, and layer thicknesses), climate classes (when possible), and pavement type (asphalt/concrete). Additional variables could have been included such as urban versus rural areas but given data availability, we limited the scope of variables. Moreover, the archetypes are limited to the production of raw materials required for the initial road construction (maintenance is not included).

Past maintenance activities have been excluded from the analysis. These include minor maintenance activities (e.g., repairing potholes or filling cracks), and rehabilitation activities (e.g., overlaying of new layers, preservation by milling the existing layer and filling with new materials, or full-depth reconstruction of the pavement). Excluding these activities prevents the estimation of past inflows/outflows of materials and their resulting GHG emissions.

Two additional limitations to our model, already highlighted in buildings studies,^60^ are the (1) overrepresentation of developed countries and (2) static approach. We developed archetypes for only two countries in Central and South America, one country in Africa and one country in Middle East and Central Asia based on data accessibility. Consequently, our model does not capture variations between countries in these regions while roads might be very different. Our analysis does not directly estimate the uncertainty using archetypes for some countries as proxy for others. It is assumed that this uncertainty is covered by the lower/upper estimates of the road width and material intensities. Roads geometric characteristics (length, width, material intensities) are static. Our model looks at roads as if they had been all built together at once while road networks are constantly being modified. The model does not capture historical patterns of material use throughout time with design and technology changes. While the number of lanes for each road type has been automatically collected for a large number of countries by using OSM, lane-width was the same for all countries (just two different ranges of lane-width depending on the road type), and material intensities were collected for a small number of countries (17 countries) due to data availability constraints such as language barrier or ease of access. The database of roads archetypes was however assumed sufficient to provide an estimate of road material stock. When it comes to the estimation of embodied GHG emissions, the emissions factors are also static in time. They do not cover the changes in material extraction, processing and transportation that happened since roads were built. These estimated emissions are likely to be lower than the historical emissions but are relevant as a reference for future road stock.^61^ The model developed in this research paper should be seen as an opportunity for further development.

To assess the importance of road material stock, we compare it with prior bottom-up material stock studies on buildings. The material stock of residential buildings in 2015 has been estimated to be around 134 Gt (ODYM-RECC model from Pauliuk et al.^62^), 270 Gt for residential buildings in 2018,^60^ and 291 Gt for residential and service buildings in 2015.^63^ For further analysis, an indicator, the Roads-to-Buildings ratio (amount of material stocked in roads divided by amount of material stocked in buildings), can be calculated to provide insights on material stocks in different parts of the built environment.^64,65^ For a few countries, we calculated Roads-to-Residential Buildings ratio (RtRB) and Roads-to-Buildings ratio (RtB) based on material stock of paved roads (this study) and material stock of buildings from existing literature^62,63^ (Table S20). Japan and China are presenting the lowest RtRB and RtB values (between 0.4 and 2.1), the United States are having the highest (between 2.7 and 4.7) while Canada, India and Europe are presenting middle range values (between 1.2 and 3.4). The values should be considered with caution due to uncertainties in the stocks, but some patterns appear and inform us on how the built environment differentiates between regions. If roads are considered as being links between buildings, Japan and China seem to have developed their built environment in a more efficient way compared to the United States as much less road material stock is needed compared to the buildings.

Urban areas are inevitably expected to expand and be more populated in the future. As road material stock scales almost linearly or superlinearly with urban surface area, alternatives to road transport to fulfill transport needs as well as different road designs are needed to reduce material use and mitigate GHG emissions. Archetypes consider variability of pavement design on a multinational level and provide a convenient framework to evaluate decarbonization strategies to reduce material use (e.g., material efficiency strategies^66^) and therefore GHG emissions. However, the entire life cycle should be considered to avoid shifting of emissions by only focusing on the raw materials for the initial construction phase.

Improving or maintaining the GRIP dataset is outside the scope of this research, but a general recommendation is to work towards better mapping of roads. The incompleteness of the paved road network data was one of the main challenges. Better maps of transport infrastructure would not only improve our results (e.g., more accurate estimation of the road length; urban areas from other regions could be included in the multivariable regression analysis) but also provide insights on how climate change impacts and will impact road infrastructure and consequently our mobility.

## Supporting Information Available

Data plotted in Figures 1, 2, 4, 5, S4, and S5, material intensity coefficients, number of lanes of road archetypes, the generated paved road length inventory, and additional details about the multivariable regression analysis presented in Table 2 (XLSX)
Methodological details and assumptions (PDF)
