# Supplementary material for: Material Stock and Embodied Greenhouse Gas Emissions of Global and Urban Road Pavement
Source: Environ Sci Technol. 2022 Dec 1;56(24):18050–9. doi: 10.1021/acs.est.2c05255 (PMC9775204; doi:10.1021/acs.est.2c05255)
Supplement: Supplementary file 2 — es2c05255_si_002.pdf [file es2c05255_si_002.pdf]

Supporting Information

Material Stock and Embodied Greenhouse Gas Emissions of Global and Urban Road Pavement

Author names

Lola S. A. Rousseau[a,*], Bradley Kloostra[b], Hessam AzariJafari[c,d], Shoshanna Saxe[b], Jeremy Gregory[e], Edgar G. Hertwich[a]

Author address

[a] Industrial Ecology Programme, Department of Energy and Process Engineering, NTNU – Norwegian University of Science and Technology, Høgskoleringen 5, 7034 Trondheim, Norway

[b] Department of Civil & Mineral Engineering, University of Toronto, 35 St. George Street, Toronto, Ontario M5S 1A4, Canada

[c] School for Environment and Sustainability, University of Michigan, Dana Building, 440 Church Street, Ann Arbor, Michigan 48109, United States

[d] Civil & Environmental Engineering, Massachusetts Institute of Technology, 77 Massachusetts Avenue, Cambridge, Massachusetts 02139, United States

[e] MIT Climate and Sustainability Consortium, Massachusetts Institute of Technology, 105 Broadway Street, Cambridge, Massachusetts 02142, United States

[*]Corresponding Author: lola.s.a.rousseau@ntnu.no

Number of pages: 43

Number of figures: 9

Number of tables: 20

Table of Contents

S1. Description of the global road network S3
S1.1. Climate conditions S3
S1.2. Length of the global road network S4
S2. Description of the road archetypes S5
S2.1. Width S5
S2.2. Cross section S7
S3. GHG emissions of road materials S10
S3.1. Asphalt S11
S3.1.1. Bitumen production S11
S3.1.2. Aggregates production S17
S3.1.3. Asphalt mixture production S21
S3.1.4. Transportation to the construction site S22
S3.1.5. GHG emissions of asphalt S23
S3.2. Granular S24
S3.3. Concrete & cement S24
S4. Limitations of GRIP paved road length S25
S5. Material stock and embodied GHGs S27
S5.1. Results per country S27
S5.2. Results per country and per capita S28
S5.3. Further analysis of United States, China, and India S29
S6. Roads in urban areas S30
S6.1. Population and GDP per urban area S31
S6.2. Urban areas included in the analysis S31
S6.3. Regression models S32
S7. National and regional studies from the literature S35
S7.1. Material stock per capita S35
S7.2. Roads-to-Buildings ratios S36

S1. Description of the global road network

The GRIP (Global Roads Inventory Project) dataset[1] provides us with a spatial representation of the global road network as an assembly of road segments. The GRIP dataset represents about 21.6 million km of roads (combining about 60 geospatial datasets of global and national road infrastructure). In the GRIP dataset, a road segment is, among other things, defined by its geometry (polyline with spatial coordinates), and attributes determining the country it is in, its type (highway, primary, secondary, tertiary, or local) and the surface type (paved, gravel, dirt/sand, steel, wood, grass, unspecified).

In existing literature, the GRIP dataset was used to evaluate roads' impact on biodiversity as part of the GLOBIO model.[2] The dataset was also applied to spatially distribute direct CO_2 emissions released by the transport sector as part of the Global Carbon Grid.[3]

As the global road network is available in a spatial representation, this enables us to get information about their local environment, more specifically, the climate conditions.

S1.1. Climate conditions

The GRIP dataset[1] is combined with a raster file of climate zones following the Köppen-Geiger climate classification[4]: climate raster values are extracted at location of the road segments' centroids. The procedure is performed in a Python environment using two Python libraries: Geopandas 0.9.0[5] and Rasterio 1.1.2[6]. When spatially joining the road segments with climate zones, some road segments are not associated with any climate zone, due to the road polyline centroids falling outside of the defined climate zones (typically in coastal areas or on small islands). The closest climate zone is therefore associated to these road segments using cKDTree spatial index from the Python library Scipy[7].

The Köppen-Geiger climate classification consists of 30 climate zones. For simplification, we aggregate them into four classes (as per the Long-Term Pavement Performance (LTPP) classification[8]) as described in Table S1.

Table S1 - Aggregation of Köppen-Geiger climate zones into four climate classes based on the LTPP classification

Climate class	Climate zones from the Köppen-Geiger climate classification
Wet, non-freeze (WN)	Af, Am, Aw, As, Cfa, Cfb
Dry, non-freeze (DN)	Bsh, BSk, BWh, Csa, Csb, Csc, Cwa, Cwb
Wet, freeze (WF)	Cfc, Dfa, Dfb, Dfc, Dfd, Dwa, Dwb
Dry, freeze (DF)	BWk, Cwc, Dsa, Dsb, Dsc, Dsd, Dwc, Dwd, EF, ET

S1.2. Length of the global road network

The polylines representing the global road network in the GRIP dataset are projected to calculate their length in meters. The projection chosen for each GRIP region is given in Table S2.

Table S2 – Projection chosen for each GRIP region

GRIP region	Projection	EPSG / ESRI code	Info
North America	North America Equidistant Conic	ESRI:102010	https://epsg.io/102010
Central and South America	South America Equidistant Conic	ESRI:102032	https://epsg.io/102032
Africa	Africa Equidistant Conic	ESRI:102023	http://epsg.io/102023
Europe	Europe Equidistant Conic	ESRI:102031	https://epsg.io/102031
Middle East and Central Asia	Asia North Equidistant Conic	ESRI:102026	http://epsg.io/102026
South and East Asia	Asia North Equidistant Conic	ESRI:102026	http://epsg.io/102026
Oceania	Australian Centre for Remote Sensing Lambert	EPSG:4462	https://epsg.io/4462

Two additional geometric characteristics are required to get a complete description of the road segments: (1) their width and (2) their cross section (thicknesses of the layers composing the pavement). As it is impossible to get this information for all the road segments of GRIP, road archetypes are applied.

S2. Description of the road archetypes

The road archetypes developed in this study are typical representation of roads according to the country, the climate class ((1) wet, non-freeze, (2) dry, non-freeze, (3) wet, freeze, and (4) dry, freeze), the road type (the five road types from GRIP: (1) highway, (2) primary, (3), secondary, (4) tertiary, and (5) local), and the pavement type (asphalt or concrete). Road type is considered to some extent a function of traffic volume. Our archetypes consist only of their material composition, energy used for their construction is out of the scope.

S2.1. Width

The typical number of lanes for each road segment is collected from OpenStreetMap (OSM) using the Python library Pyrosm[9]. OSM is an open-source geographic database built on a collaborative effort of users across the globe and is an easy solution to get geometric characteristics of roads in a large panel of countries. The attribute "number of lanes" is chosen over the attribute "width" as more road segments in OSM have this attribute and would constitute a better sample for estimating width of road in a later stage. Dual carriageways in OSM are mapped as two parallel lines while in GRIP, they are mapped as one line to avoid double counting of the road length. For road types "motorway", "primary", "trunk" and "secondary" in OSM, their number of lanes is multiplied by 2 if they are tagged as being "oneway". It is assumed that "motorway", "primary", "trunk" and "secondary" roads which are "oneway" are part of a dual carriageway. Road types from OSM are then mapped to the road type from GRIP as shown in Table S3 obtained from Meijer et al.[1]. Lastly, weighted averages of number of lanes by country and road type are calculated.

Table S3 – Mapping between GRIP road types and OpenStreetMap road types

Road types from GRIP	Road types from OpenStreetMap
Highway	motorway, motorway_link
Primary	trunk, trunk_link, primary, primary_link
Secondary	secondary, secondary_link
Tertiary	tertiary, tertiary_link
Local	residential, living_street

In a second stage, the width is estimated by multiplying the number of lanes with a range of lane widths according to Table S4.

Table S4 - Width of road segments (width low estimate, width high estimate) by road type

**Road type**	**Width – low estimate**	**Width – high estimate**
Highway, primary	Lane count × 3.5 m	(Lane count + 1) × 4 m
Secondary, tertiary, local	Lane count × 3 m	(Lane count + 1) × 3.5 m

Table S5 presents low and high estimates for the lane width for a selected number of countries in each GRIP region allowing us to define the lower and upper range values described above. Choosing lower and higher bounds allow us to also consider partly that there might be shoulders on the side of the roads, additional emergency lanes, and exit/entrance auxiliary lanes.

Table S5 – Lane widths (from design codes or from literature)

**GRIP region**	**Country and references**	**Road type**	**Low estimate**	**High estimate**
North America	USA[10]	Highway & Primary	3	3.7
		Secondary, Tertiary & Local	2.4	3.7
	Canada[11]	Highway & Primary	3.5	3.7
		Secondary, Tertiary & Local	3	3.7
Central and South America	Chile[12]	Highway & Primary	3.5	4
		Secondary, Tertiary & Local	2.75	3.25
Africa	South Africa[13]	Highway & Primary	3.7	3.7
		Secondary, Tertiary & Local	2.7	3.4
Europe	Typical European values[14,15]	Highway & Primary	3.5	3.75
		Secondary, Tertiary & Local	3	3.5
	Spain[16]	Highway & Primary	3.5	3.5
		Secondary, Tertiary & Local	3	3.5
	Great Britain[17,18]	Highway & Primary	3.65	3.7
		Secondary, Tertiary & Local	3	4.1
	Germany[19,20]	Highway & Primary	3.5	3.75
		Secondary, Tertiary & Local	3.25	3.5
Middle East and Central Asia	The United Arab Emirates[21]	Highway & Primary	3.65	3.65
		Secondary, Tertiary & Local	3.65	3.65
South and East Asia	China[22]	Highway & Primary	3.5	3.75
		Secondary, Tertiary & Local	3.5	3.75
	Japan[23]	Highway & Primary	3	3.75
		Secondary, Tertiary & Local	2.75	3.5
	India[24]	Highway & Primary	3.5	3.5
		Secondary, Tertiary & Local	3.5	3.5
	Vietnam[25]	Highway & Primary	3.75	3.75
		Secondary, Tertiary & Local	3	3.5
Oceania	Australia[26] (applicable to New Zealand)	Highway & Primary	3.5	3.5
		Secondary, Tertiary & Local	3.2	3.5

S2.2. Cross section

Archetypes represent the pavement layers and thicknesses at age = 0 (the initial construction of the road). During its lifetime, each road segment undergoes maintenance treatment such as overlays or reconstruction. Therefore, the cross sections used to represent typical roads may not correspond to the reality of the current road stock. However, this still enables us to provide an estimate of the global road material stock.

Two pavement types are considered: flexible pavement and rigid pavement. The pavement consists generally of three layers: subbase course, base course, and surface course. On one hand, the flexible pavement has the surface course (and possibly the base course) made of asphalt*. On the other hand, rigid pavement is made of concrete. The base and subbase courses are usually composed of granular materials alone or with binding substances. There may be a large panel of configurations in the composition of the different pavement layers. For the sake of consistency and simplicity in collecting and organizing the data, it is assumed that the pavement consists of two layers: a layer of asphalt (flexible pavement) or a layer of concrete (rigid pavement) on top of a layer of granular materials (which can be bound with cement in some countries).

Due external constraints (as language barrier or ease of access), it was not possible to collect cross section data for all the countries available in the GRIP dataset. Therefore, the data collection was limited to a few countries in each GRIP region and derivations of archetypes for these countries were used as proxies for the other countries in the same region. The countries for which data were collected are presented in Table S6. If possible, data were collected to fit the road types from GRIP (highway, primary, secondary, tertiary, and local roads) and the four climate classes (WN, DN, WF, DF). When

* There often are issues of vocabulary about asphalt; in this paper, asphalt (sometimes called “asphalt concrete”) is considered as the mix of granular material, bitumen, and potential additives.

it was not possible to find data disaggregated into climate class, the same values were used for all the road segments independently of their climate conditions.

To sum up, the data collected are the following:

- More than 830 flexible pavement designs (for Canada, European countries, Chile, Mexico, South Africa, India, New Zealand, and Australia).
- 320 rigid pavement designs (Canada, European countries, Chile, and Mexico).
- Minimum layer thicknesses for the United Arab Emirates.
- Archetypes retrieved from scientific literature for China, Japan, and Vietnam.

Table S6 – Countries for which material intensity data have been collected

GRIP region	Country and references	Comments
North America	USA[27,28]	Thickness of the surface course from Gregory et al.[27]. The thickness of the granular layer is then inferred using a linear regression based on LTPP data[28].<br>Lower/Upper estimates inferred based on Canada's lower/upper estimates
	Canada[29–33]	Lower (35th percentile) – Upper (65th percentile) – Median estimates calculated based on the set of pavement designs collected.
Central and South America	Chile[34]	Lower (35th percentile) – Upper (65th percentile) – Median estimates calculated based on the set of pavement designs collected.
	Mexico[35]	Lower (35th percentile) – Upper (65th percentile) – Median estimates calculated based on the set of pavement designs collected.
Africa	South Africa[36,37]	Lower (35th percentile) – Upper (65th percentile) – Median estimates calculated based on the set of pavement designs collected.
Europe	Germany[38]	Lower (35th percentile) – Upper (65th percentile) – Median estimates calculated based on the set of pavement designs collected.
	Austria[39]	Lower (35th percentile) – Upper (65th percentile) – Median estimates calculated based on the set of pavement designs collected.
	Spain[40]	Lower (35th percentile) – Upper (65th percentile) – Median estimates calculated based on the set of pavement designs collected.
	Italy[41]	Lower (35th percentile) – Upper (65th percentile) – Median estimates calculated based on the set of pavement designs collected.

	Great Britain[17]	Lower (35th percentile) – Upper (65th percentile) – Median estimates calculated based on the set of pavement designs collected.
Middle East and Central Asia	The United Arab Emirates[42]	Based on minimum values, then median and max inferred from Australian's data (the Pavement Design Manual[42] mentions that the design procedures are based on AASHTO and Austroads).
South and East Asia	China[22]	Archetypes taken from scientific literature. Lower/Upper inferred based on India's lower/upper estimates.
	Japan[43]	Archetypes taken from scientific literature. Lower/Upper inferred based on India's lower/upper estimates.
	India[44]	Lower (35th percentile) – Upper (65th percentile) – Median estimates calculated based on the set of pavement designs collected.
	Vietnam[45]	Archetypes taken from scientific literature. Lower/Upper inferred based on India's lower/upper estimates.
Oceania	Australia[46]	Lower (35th percentile) – Upper (65th percentile) – Median estimates calculated based on the set of pavement designs collected.
	New Zealand[47]	Granular layer calculated based on Australia's to get the same total pavement thickness. Lower (35th percentile) – Upper (65th percentile) – Median estimates of asphalt layer thickness calculated based on the set of pavement designs.

The layer thicknesses are in a next step converted to material intensities (in kg/m^2) using their respective density collected from the database available in the software Athena Pavement LCA[48] as averages of the products available in their database – 2.3x10^3 kg/m^3 for asphalt, 2.4x10^3 kg /m^3 for granular, 2.3x10^3 kg /m^3 for concrete and 3.15x10^3 kg/m^3 for cement. If the granular materials are bound with cement, the ratio of cement is assumed to be 3%[49] by volume (ratio of cement from South Africa[49] but assumed to be applicable to other regions).

In the GRIP dataset, the surface type does not specify if the road is paved with asphalt or concrete. Therefore, the material intensities of flexible pavement and rigid pavement were combined into single material intensities by applying ratios of flexible versus rigid pavements in each country (and by road type if available). Table S7 provides the references used to determine the ratios.

Table S7 - Ratios flexible versus rigid pavement – sources of data

GRIP region	Country and references	Comments
North America	USA[50]	Shares of flexible pavement and rigid pavement are calculated based on their roadway length in the country (i.e.) 70% highway are flexible, 92% primary roads are flexible, 97% secondary and tertiary roads are flexible, and 100% local roads are flexible.
	Canada[51]	Assuming local roads 100% asphalt. The other road types are 68% flexible pavement (leading to 90% of roads being of surfaced with asphalt as stated by EAPA[51]).
Central and South America	Chile	Assuming same ratios same as Mexico.
	Mexico[51]	Assuming local roads 100% asphalt. The other road types are 96% flexible.
Africa	South Africa	Assuming 100% flexible pavement.
Europe	Germany[52]	25% of highways are rigid pavement.<br>Assuming all other road types are 100% flexible pavement.
	Austria[52]	Two-thirds of surface highways made of concrete (since all highways same archetypes, assumed two-thirds of the length).<br>Assuming all other road types are 100% flexible pavement.
	Spain	Own assumption: 90% asphalt except local roads 100% asphalt.
	Italy	Own assumption: 90% asphalt except local roads 100% asphalt.
	Great Britain[53]	80% of highways are flexible pavement and all the other road types are 95% flexible pavement (except local roads 100% flexible – own assumption).
Middle East and Central Asia	The United Arab Emirates	Own assumption: 90% asphalt except local roads 100% asphalt.
South and East Asia	China	Assuming 100% flexible pavement.
	Japan[54]	Shares of flexible pavement and rigid pavement are calculated based on their roadway length in the country (i.e.) 93% of highways are flexible, 96% of primary roads are flexible, 98% of secondary and tertiary roads are flexible, and 74% of local roads are flexible.
	India	Assuming 100% flexible pavement.
	Vietnam	Assuming 100% flexible pavement.
Oceania	Australia	Assuming 100% flexible pavement.
	New Zealand	Assuming 100% flexible pavement.

S3. GHG emissions of road materials

Material stocks vary regionally, but the GHG emissions per unit of material is also subject to regional variations. Therefore, instead of using a single value of carbon intensity per unit of material, we

develop region-specific values. Only GHG emissions resulting from the production of road materials are considered.

S3.1. Asphalt

Asphalt is the mixture of bitumen (asphalt binder), aggregates and potentially additional substances (such as fly ash or hydrated lime[55]). The GHG emissions are accounted from "well-to-construction site" i.e., from the production of raw materials to produce asphalt until the transportation to the construction site. Table S8 provides details on the processes included in the modeling of asphalt production.

Table S8 – Details on modeling of asphalt production

Phase of asphalt production	Processes	Inputs
Materials production	Bitumen production	Crude oil (see Sections S3.1.1.1 and S3.1.1.2) + Other inputs from Ecoinvent v3.6[56] (see Section S3.1.1.3)
	Aggregates production	Crushed gravel from Ecoinvent v3.6[56] (see Section S3.1.2)
Asphalt mixture production	Energy use for heating/mixing	Energy use based on processes from Ecoinvent v3.6 (see Section S3.1.3)
Transportation of asphalt mixture to the construction site	Transportation	Lorry transport from Ecoinvent v3.6[56] (see Section S3.1.4)

S3.1.1. Bitumen production

The bitumen production is divided into three stages: (1) crude oil extraction, (2) crude oil trade and consumption, and (3) bitumen production. Data collection and processing are described in the following sections.

S3.1.1.1. Crude oil extraction

Crude oil is a natural nonrenewable resource composed of hydrocarbons. It is extracted to be refined into petroleum products such as gasoline, kerosene, or diesel fuel. The carbon intensity of crude oil presents large variations depending on the crude oil density as well as extraction and processing methods.[57]

Crude oil extraction is responsible for most of the greenhouse gases (GHGs) emitted by bitumen production: its contribution ranges from 50% to 63% according to the Life Cycle Assessment performed for the Asphalt Institute[58] and is around 70% of the bitumen global warming potential estimated by Eurobitume[59]. The importance of building a model that would estimate the carbon intensity of crude oil consumed for bitumen production is thus emphasized.

Masnadi et al.[57] evaluates the carbon intensity of crude oil from well-to-refinery gate (exploration, drilling, extraction, processing, and transport to the refinery). This study provides the average, the 5th and the 95th percentiles of crude oil carbon intensity (gCO_2-eq/MJ crude oil) for 90 countries. About 98% of crude oil produced in 2015 is covered. Only one year is available, but it is assumed that this year is representative of crude oil extraction activities even if external factors might disrupt oil supply[60] and influence the production.

Instead of using the average value of crude oil carbon intensity, we chose to reproduce its probability density function. Most of the inputs of crude oil production model developed by Masnadi et al.[57] follow a lognormal distribution and lead to skewed 5th and 95th percentiles. It is thus assumed that lognormal would be an appropriate distribution. A least squares optimization procedure is applied to find, for each producing country, a lognormal distribution that would fit best the average, 5th and 95th percentiles. Five countries (Indonesia, Kyrgyzstan, Bulgaria, Romania, Spain) have a relative error of their fitted average of more than 20% (up to 59% for Indonesia). Indonesia, Kyrgyzstan, Bulgaria, and Romania have a relative error of their fitted 5th percentile of more than 20%. No country has a relative error of their fitted 95th percentile of more than 20%. The fitted average, 5th and 95th percentiles for the other countries are considered reasonable.

S3.1.1.2. Crude oil trade and consumption

It is extremely difficult to determine how much crude oil extracted by a country is processed, exported, and further transformed (e.g., into bitumen) in another country to be used by this country or to be exported in another one. Crude oil can be imported and re-exported without any transformation; it can be imported, processed, and used/exported; refined products can also be directly imported.[61,62]

We nevertheless built a simple model of crude oil consumption (from own production and from imports) based on two databases (period of analysis: 2010-2015):

- (1) production, imports, and exports of crude oil from the International Energy Agency (IEA)[63].
- (2) the International Trade Database at the product-level (BACI) from French research institute CEPII[64].

The BACI database has limitations (erroneous report of trade information, aggregation of several products, trading partners and quantities might not be fully reported due to confidentiality, etc.)[65] but it is to our knowledge the most comprehensive publicly available source of data to identify trade of crude oil adapted to our modeling. Crude oil refining is a complex process for which carbon intensities by country and crude oil type have been calculated[66] without identifying carbon intensity of crude oil refining specifically for bitumen production.

Here we decide to build a simple model of crude oil trade and consumption relying on a few strong assumptions which have their limits but are necessary in the light of data availability. The assumptions are described throughout the model along with their respective equations. The model's variables and their description are presented in Table S9.

Table S9 – Variables of the crude oil trade and consumption model with their description

Variables	Description
$prod$	Production of crude oil
exp	Exports of crude oil
$\exp_{imp}$	Exports of crude oil from imports (only stored in the country)
imp	Imports of crude oil
$cons$	Consumption total of crude oil by a country
$cons_{own}$	Consumption of crude oil from own production
$cons_{imp}$	Consumption of crude oil resulting from imports

Equation (1) shows the situation in which production is larger than exports. It is assumed countries export their own production and consume what is left from their own production. In addition, it is assumed that no exports are originally from imports.

$$if\ prod \geq exp: \begin{cases} cons_{own} = prod - exp \\ \exp_{imp} = 0 \end{cases}$$ Equation (1)

This assumption would not consider countries which have a larger production than their exports but would still import and later re-export.

If production is strictly smaller than exports (either production is null – seven countries in this situation, or it is not null but smaller than exports – only three countries in this situation), it is assumed the consumption from domestic production is null and we can calculate the export of crude oil from imports as shown in Equation (2).

$$if\ prod < exp: \begin{cases} cons_{own} = 0 \\ \exp_{imp} = exp - prod \end{cases}$$ Equation (2)

The consumption from imports can therefore be calculated according to Equation (3).

$$cons_{imp} = imp - \exp_{imp}$$ Equation (3)

The total consumption of crude oil is thus the sum of consumption from domestic production and from imports (Equation (4)).

$$cons = cons_{own} + cons_{imp}$$ Equation (4)

We test the results of our model to check if we have:

$$\begin{cases} cons_{own} \geq 0 \\ cons_{imp} \geq 0 \\ cons \geq 0 \end{cases}$$ Equation (5)

For the period 2010-2015, only one country (Democratic Republic of the Congo) seems to export more than they produce while they do not have any import. This leads to negative consumption of crude oil. This could be due to missing or erroneous data. We choose to drop the country from the model.

We calculate the ratios of crude oil consumption from own production to crude oil consumption from imports in Equation (6).

$$\begin{cases} ratio(cons_{own}) = \frac{\mathrm{cons_{own}}}{\mathrm{cons}} \\ ratio(cons_{imp}) = \frac{cons_{imp}}{cons} \end{cases} \qquad \text{Equation (6)}$$

We want to disaggregate the consumption of imported crude oil into its origins (countries of extraction) using the BACI database providing details about imports and exports of crude oil per country (product code: 270900)[64]. However, we first calculate the total exports and imports of crude oil per country based on BACI data. We keep the production values from IEA and apply the model as described in Equations (1), (2), (3), (4), (5), and (6).

This way, we can compare the ratios of crude oil consumption from own production to crude oil consumption from imports obtained from IEA data and obtained using BACI+IEA dataset. For countries where only ratios calculated in the IEA model are available, we keep them. For the other countries, we keep the values from BACI if the sum of the square of difference of the ratios is lower than 0.1. Lastly, we remove the countries for which the ratios between IEA and BACI+IEA are too different as discrepancies in the data are highlighted (only three countries fall into this category: Cuba, Kyrgyzstan, and Syria).

Results of our model provide a disaggregation of crude oil consumption by country into crude oil consumption resulting from own production of crude oil and from imports. Figure S1 presents the ratio of crude oil consumption for a selected set of countries.

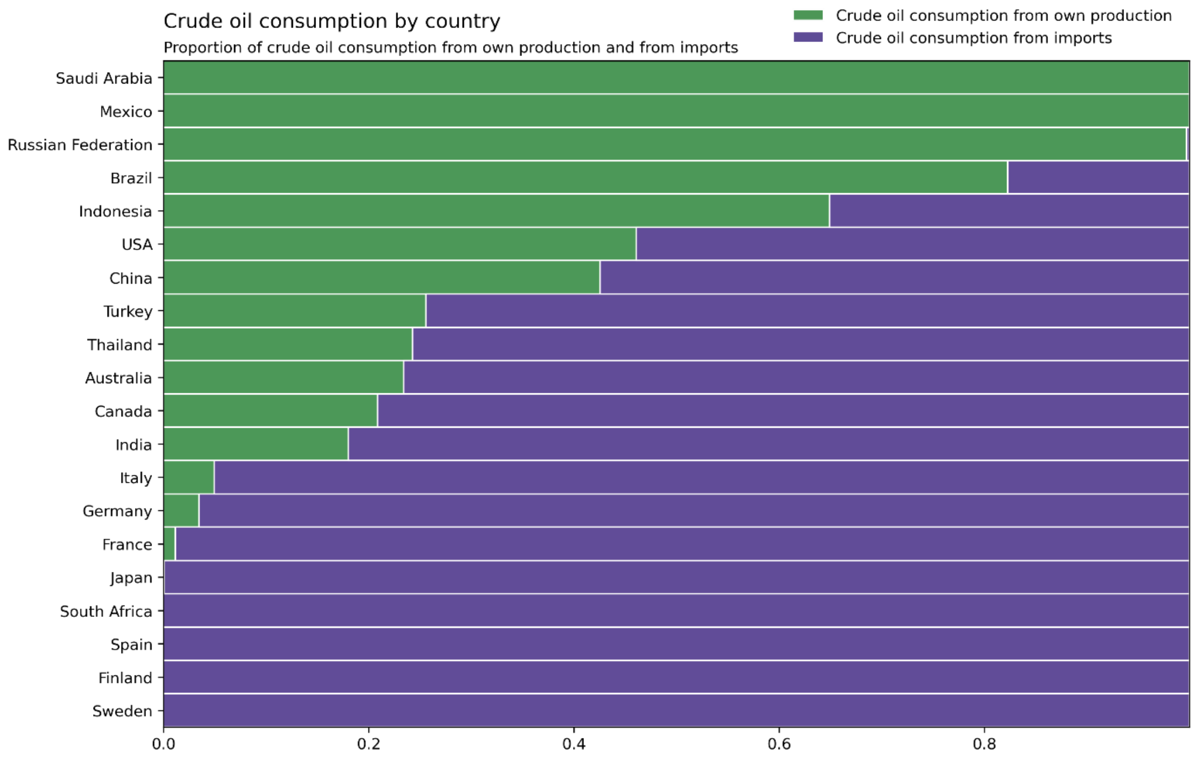

Figure S1 – Crude oil consumption by country (disaggregated into crude oil consumption from own production and crude oil consumption from imports)

In a second stage, BACI database is used to disaggregate the imports of crude oil by the country of extraction (crude oil extracted transported through several countries cannot be tracked and therefore the import by the destination defines the extraction).

Even if a model for trade of crude oil has been built and identifies in a simple way from where crude oil has been extracted to where it has been transported, nothing is done to change the transport. Masnadi et al.[57] already includes an average transport from well to refinery gate and argues that transport does not have a large contribution to the GHG emissions.

Lastly, the GHG emissions per MJ of crude oil consumed by a country is calculated multiplying the ratios of domestic crude oil and imported crude oil (disaggregated into the countries where it comes from) with the GHG emissions from crude oil extraction.

S3.1.1.3. Other inputs in bitumen production

These calculated GHG emissions per MJ of crude oil consumed are used to replace the GHG emissions of petroleum in pitch production process (assumed to be representative of bitumen production) from Ecoinvent v3.6[56]. The pitch production process is available for a few regions. One pitch production process is associated with a GRIP region (which will be applied to the countries located in the respective region) to estimate the GHG emissions from other activities in the production of bitumen.

Table S10 – Ecoinvent v3.6 processes used to determine other inputs in bitumen production

GRIP region	Ecoinvent v3.6 process, pitch production
North America	pitch production, petroleum refinery operation, RoW
Central and South America	pitch production, petroleum refinery operation, BR
Africa	pitch production, petroleum refinery operation, ZA
Europe	pitch production, petroleum refinery operation, Europe without Switzerland
Middle East and Central Asia	pitch production, petroleum refinery operation, RoW
South and East Asia	pitch production, petroleum refinery operation, IN
Oceania	pitch production, petroleum refinery operation, RoW
BR: Brazil; IN: India; RoW: Rest-Of-World; ZA: South Africa	

Arda, a MATLAB-excel tool for LCA calculations, developed in the Industrial Ecology programme at NTNU[67] is used to modify the Ecoinvent processes and calculate the GHG emissions using ReCiPe Midpoint (H) V1.13 with allocation, cut-off by classification.

GHG emissions for bitumen production are thus estimated for all the countries we have been able to calculate the ratios of crude oil consumption from own production and imports. Our model might result in countries refining crude oil to produce bitumen while this might not be the case. However, it is assumed that if countries are reported to produce and/or import crude oil, they have refining facilities and would therefore produce bitumen.

S3.1.2. Aggregates production

The impact of aggregates is estimated based on crushed gravel activity from Ecoinvent v3.6[56]. The crushed gravel markets available in the database consist of crushed gravel production and transport. For each GRIP region, a crushed gravel market is selected and modified to be a generic representation

of the activity in the region. When possible, the market was also adapted to fit one or more countries located in the region. The modifications are made on the electricity used in the crushed gravel production and on the transport specified in the market activity as specified in Table S11. The transport distance is however unchanged. Calculations are performed with Arda (Allocation, cut-off by classification and use of ReCiPe Midpoint (H) V1.13).

Table S11 – Modifications of market for gravel, crushed from Ecoinvent v3.6

GRIP region	Country	Original market activity from Ecoinvent v3.6	New electricity process in “gravel production, crushed”	New transportation processes in “market for gravel, crushed”
North America	Canada	market for gravel, crushed, CA-QC	Not modified	Not modified
	USA	market for gravel, crushed, CA-QC	market group for electricity, medium voltage, US	market for transport, freight train, US<br>market for transport, freight, light commercial vehicle, RoW<br>market for transport, freight, lorry, unspecified, RoW
	Generic	market for gravel, crushed, CA-QC	market group for electricity, medium voltage, RNA	market for transport, freight train, US<br>market for transport, freight, light commercial vehicle, RoW<br>market for transport, freight, lorry, unspecified, RoW
Central and South America	Brazil	market for gravel, crushed, BR	Not modified	Not modified
	Generic	market for gravel, crushed, BR	market group for electricity, medium voltage, RLA	Not modified
Africa	South Africa	market for gravel, crushed, RoW	market for electricity, medium voltage, ZA	market for transport, freight train, ZA<br>market for transport, freight, light commercial vehicle, ZA<br>market for transport, freight, lorry, unspecified, ZA
	Generic	market for gravel, crushed, RoW	market group for electricity, medium voltage, RAF	market for transport, freight train, ZA<br>market for transport, freight, light commercial vehicle, ZA<br>market for transport, freight, lorry, unspecified, ZA
Europe	France	market for gravel, crushed, CH	market for electricity, medium voltage, FR	market for transport, freight train, Europe without Switzerland<br>market for transport, freight, lorry, unspecified, RER
	Spain	market for gravel, crushed, CH	market for electricity, medium voltage, ES	market for transport, freight train, Europe without Switzerland<br>market for transport, freight, lorry, unspecified, RER
	Germany	market for gravel, crushed, CH	market for electricity, medium voltage, DE	market for transport, freight train, Europe without Switzerland<br>market for transport, freight, lorry, unspecified, RER
	Sweden	market for gravel, crushed, CH	market for electricity, medium voltage, SE	market for transport, freight train, Europe without Switzerland<br>market for transport, freight, lorry, unspecified, RER

	Generic	market for gravel, crushed, CH	market for electricity, medium voltage, RER	market for transport, freight train, Europe without Switzerland<br>market for transport, freight, lorry, unspecified, RER
Middle East and Central Asia	Russia	market for gravel, crushed, RoW	market for electricity, medium voltage, RU	Not modified
	Generic	market for gravel, crushed, RoW	market group for electricity, medium voltage, RME	Not modified
South and East Asia	China	market for gravel, crushed, IN	electricity, medium voltage / market group for electricity, medium voltage / CN / kWh	Same transport as market for gravel, crushed, RoW
	Japan	market for gravel, crushed, IN	electricity, medium voltage / market for electricity, medium voltage / JP / kWh	Same transport as market for gravel, crushed, RoW
	India	market for gravel, crushed, IN	Not modified	Not modified
	Generic	market for gravel, crushed, IN	electricity, medium voltage / market group for electricity, medium voltage / RAS / kWh	Same transport as market for gravel, crushed, RoW
Oceania	Australia	market for gravel, crushed, RoW	market for electricity, medium voltage, AU	Not modified
	New Zealand + Generic	market for gravel, crushed, RoW	market for electricity, medium voltage, NZ	Not modified

AU: Australia; BR: Brazil; CA-QC: Quebec (Canada); CH: Switzerland; CN: China; DE: Germany; ES: Spain; FR: France; IN: India; JP: Japan; NZ: New Zealand; RAF: Africa; RAS: Asia and the Pacific; RER: Europe; RLA: Latin America and the Caribbean; RME: Middle East; RNA: North America; RoW: Rest-Of-World; RU: Russia; SE: Sweden; US: United States; ZA: South Africa

S3.1.3. Asphalt mixture production

Asphalt mixture is composed of bitumen, natural aggregates, and potentially additives. Bitumen represents about 4 to 6 % of the mixture weight[51]. Additives are ignored in our study. In addition, only virgin materials are used. The quantity of bitumen is chosen between 4 and 6% according to a uniform distribution and the quantity of crushed gravel is calculated based on the bitumen quantity so that the sum is 100%.

The average energy consumption is taken from the Environmental Guidelines on Best Available Techniques for the Production of Asphalt Paving Mixes[68] which is ranging between 270 and 390 MJ/tonne of asphalt. Any means to reduce the energy consumption such as the production of warm mix asphalt are excluded from the model.

Two types of energy are using at the asphalt plant: fossil fuel and electricity[69]. Determining a typical energy mix used in the asphalt plant is challenging. In the US, natural gas is used to power 70-90% of the asphalt plant while fuel oil is used for the other plants[70]. In several studies, asphalt plant was modeled as consuming fuel oil or diesel: in Spain[71], in Sweden[72], in Italy[73], in Singapore[74], in Australia[75], and in Colombia[76]. In Chile[77], in United Arab Emirates[78], and in another Italian case[79], it is natural gas which is powering the asphalt plant. In Austria, coal is also reported as powering the analysed asphalt plant.[80]. Some of the studies also report the use of electricity in the asphalt production (ranging from 0.3%[71] up to 10.9%[72] when reported). A complete overview on asphalt plants would be required to define a typical energy mix by country or by region. However, this is considered out of the scope of this study. Therefore, rough assumptions are made: the use of fossil fuel ranges from 95% to 99% of the energy use (electricity completes the energy use) and fossil fuel used is a mixed of fuel oil and natural gas (fuel oil ranging from 0 to 100% and natural gas completing). The two parameters follow uniform distributions.

The GHG emissions of each energy type are taken from Ecoinvent v3.6[56] - ReCiPe Midpoint (H) V1.13. The GHG emissions of energy are modeled by GRIP region. The processes considered are listed in Table S12, Table S13, and Table S14.

Table S12 – Ecoinvent v3.6 processes to model electricity

GRIP region	Ecoinvent v3.6 processes
North America	market group for electricity, medium voltage, RNA
Central and South America	market group for electricity, medium voltage, RLA
Africa	market group for electricity, medium voltage, RAF
Europe	market group for electricity, medium voltage, RER
Middle East and Central Asia	market group for electricity, medium voltage, RME
South and East Asia	market group for electricity, medium voltage, RAS
Oceania	market for electricity, medium voltage, AU
AU: Australia; RAF: Africa; RAS: Asia and the Pacific; RER: Europe; RLA: Latin America and the Caribbean; RME: Middle East; RNA: North America	

Table S13 – Ecoinvent v3.6 processes to model the use of fuel oil

GRIP region	Ecoinvent v3.6 processes
North America	heat production, light fuel oil, at industrial furnace 1MW, CA-QC
Central and South America	heat production, light fuel oil, at industrial furnace 1MW, RoW
Africa	heat production, light fuel oil, at industrial furnace 1MW, RoW
Europe	heat production, light fuel oil, at industrial furnace 1MW, Europe without Switzerland
Middle East and Central Asia	heat production, light fuel oil, at industrial furnace 1MW, RoW
South and East Asia	heat production, light fuel oil, at industrial furnace 1MW, RoW
Oceania	heat production, light fuel oil, at industrial furnace 1MW, RoW
CA-QC: Quebec (Canada); RoW: Rest-Of-World	

Table S14 – Ecoinvent v3.6 processes to model the use of heat

GRIP region	Ecoinvent v3.6 processes
North America	market for heat, district or industrial, natural gas, CA-QC
Central and South America	market for heat, district or industrial, natural gas, RoW
Africa	market for heat, district or industrial, natural gas, RoW
Europe	market group for heat, district or industrial, natural gas, RER
Middle East and Central Asia	market for heat, district or industrial, natural gas, RoW
South and East Asia	market for heat, district or industrial, natural gas, RoW
Oceania	market for heat, district or industrial, natural gas, RoW
CA-QC: Quebec (Canada); RoW: Rest-Of-World	

S3.1.4. Transportation to the construction site

The transport to the road construction site is reported ranging between 30 and 80 km[51]. A shorter distance has also been reported[81]. Therefore, a range of 10-80 km is considered, being uniformly distributed. The transport is assumed to be carried out by truck (even if in some countries, asphalt might be transported by other transport means, e.g., in Norway, where it can be transported by boat[82]).

The GHG emissions per tonne-km of asphalt transport are based on modified Ecoinvent v3.6[56] processes or other sources as described in Table S15.

Table S15 - Ecoinvent v3.6 processes to model transport

GRIP region	Ecoinvent v3.6 processes or other source
North America	market for transport, freight, lorry 16-32 metric ton, EURO5, RoW
Central and South America	transport, freight, lorry 16-32 metric ton, EURO5, RoW – modified to low-sulfur diesel only from Brazil, Colombia, and Peru (same methodology as Grael et al.[83])
Africa	market for transport, freight, lorry 16-32 metric ton, EURO2, ZA
Europe	market for transport, freight, lorry 16-32 metric ton, EURO5, RER
Middle East and Central Asia	market for transport, freight, lorry 16-32 metric ton, EURO2, ZA (Euro II trucks are the most common in this region[78])
South and East Asia	transport, freight, lorry 16-32 metric ton, EURO5, RoW – modified to low-sulfur diesel only from India. The resulting value fits in the range given by Mao et al.[84] for a medium-heavy diesel truck in China.
Oceania	Articulated truck in Australia[85]
RER: Europe; RoW: Rest-Of-World; ZA: South Africa	

S3.1.5. GHG emissions of asphalt

Figure S2 presents the GHG emissions of asphalt production for a few countries calculated after a Monte Carlo analysis of 1000 iterations.

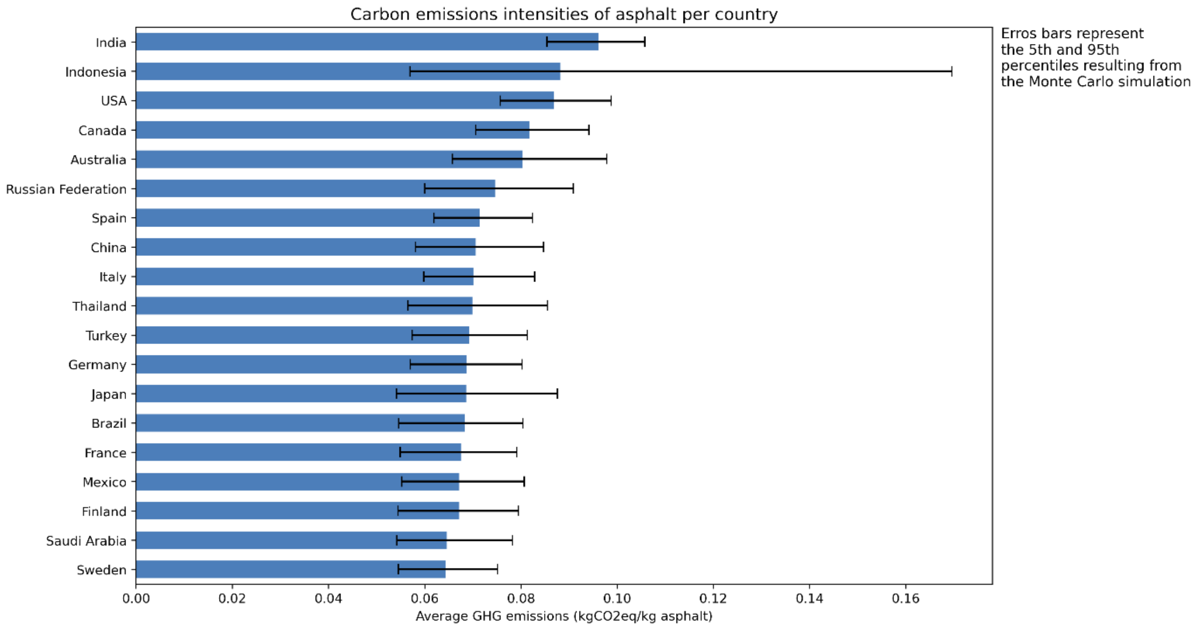

Figure S2 – Average GHG emissions of asphalt production (kgCO$_2$-eq/kg asphalt)

Summary of the sources of uncertainty in the asphalt production model:

- GHG emissions from crude oil extraction: log-normal distribution

- Quantity of bitumen & quantity of aggregates (the sum is equal to 100%): uniform distribution
- Energy quantity for asphalt production: uniform distribution
- Energy mix (fossil fuel (light fuel oil/natural gas) and electricity): uniform distributions
- Transport distance: uniform distribution

S3.2. Granular

The GHG emissions for granular materials are taken from aggregates described in Section S3.1.2.

S3.3. Concrete & cement

Concrete is modeled using the "market for concrete, normal" as shown in Table S16.

Table S16 – Ecoinvent v3.6 processes to model concrete production

GRIP region	Ecoinvent v3.6 processes
North America	market for concrete, normal, RNA
Central and South America	market for concrete, normal, BR<br>market for concrete, normal, CO<br>market for concrete, normal, PE<br>Scaled using their respective quantity in the process "road construction, RoW".
Africa	market for concrete, normal, ZA
Europe	market for concrete, normal, CH
Middle East and Central Asia	market for concrete, normal, RoW
South and East Asia	market for concrete, normal, IN
Oceania	market for concrete, normal, RoW
BR: Brazil; CH: Switzerland; CO: Columbia; IN: India; PE: Peru; RNA: North America; RoW: Rest-Of-World; ZA: South Africa	

Regarding cement, used as a binding component of aggregates in some regions, its use is modelled by the production of Portland cement as described in Table S17. Carbon intensities are collected for all the GRIP region, but cement is not necessarily appearing in the material intensities for countries in all regions.

Table S17 - Ecoinvent v3.6 processes to model cement production

GRIP region	Ecoinvent v3.6 processes
North America	market for cement, Portland, US (used as a proxy for other countries in North America except Canada)<br>market for cement, Portland, CA-QC
Central and South America	market for cement, Portland, PE<br>market for cement, Portland, BR<br>The two markets are combined into one generic value for the whole region using their respective concrete quantity in the process "road construction, RoW"
Africa	market for cement, Portland, ZA
Europe	market for cement, Portland, Europe without Switzerland
Middle East and Central Asia	market for cement, Portland, RoW
South and East Asia	market for cement, Portland, IN
Oceania	market for cement, Portland, RoW
BR: Brazil; CA-QC: Quebec (Canada); IN: India; PE: Peru; RoW: Rest-Of-World; US: United States; ZA: South Africa	

S4. Limitations of GRIP paved road length

Meijer et al.[1] acknowledges the limitations of the GRIP dataset and the issues of comparing road length calculated with the GRIP dataset with other road statistics. There are several methodologies in measuring road length leading to very different results (how to measure dual carriageways, bi-directional roads, etc.). However, as we intend to provide an estimate of the embodied material quantity and GHG emissions from road construction, limiting our paved road length to the GRIP dataset would lead to a large underestimation of material quantities and GHG emissions. In addition, availability of road surface type varies greatly among countries (for example, roads in China all have the attribute "Unspecified" as road surface or less than half of roads in the US are reported as being paved as shown on Figure S3).

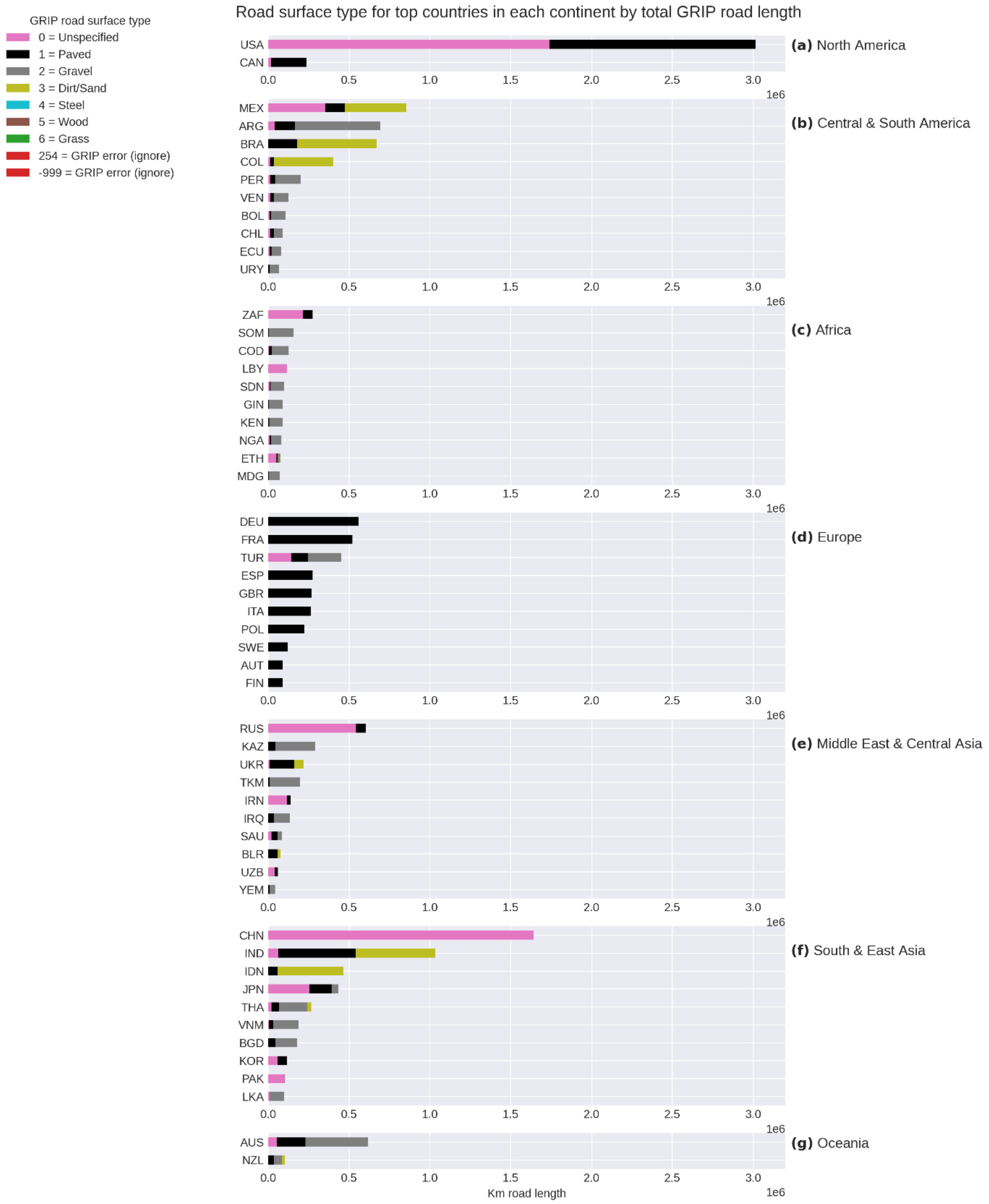

Figure S3 – Road surface type for top countries in each continent by total GRIP road length

This heterogeneity in the dataset would bias the analysis when comparing countries. Therefore, we performed a re-estimation of the road length with publicly available statistics as described in Section 2.2 from the manuscript. In fine, this allows us to understand the extent to which GRIP covers the material and GHGs embodied in the global road network.

S5. Material stock and embodied GHGs

S5.1. Results per country

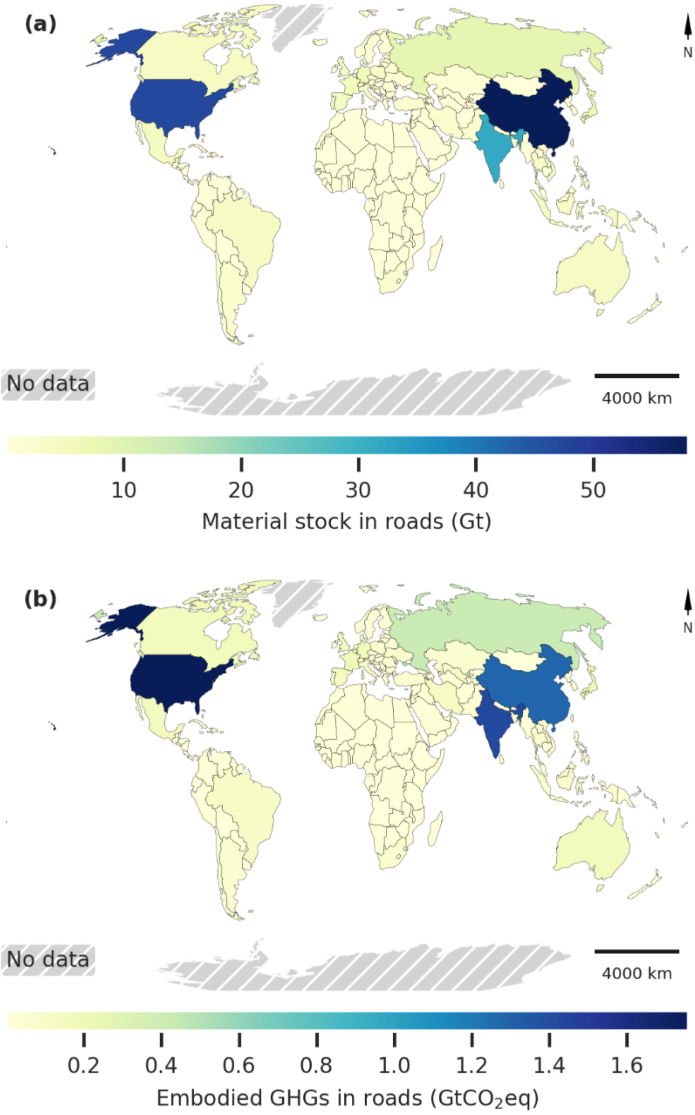

Figure S4 - On top, Figure (a) represents the material stock (Gigatonnes) in the road network per country. On the bottom, Figure (b) represents embodied GHGs (Gigatonnes CO2-eq) in the road network per country. Material stock and embodied GHGs are based on the combination of GRIP dataset with CIA World Factbook[86] and other statistics[87–94].

S5.2. Results per country and per capita

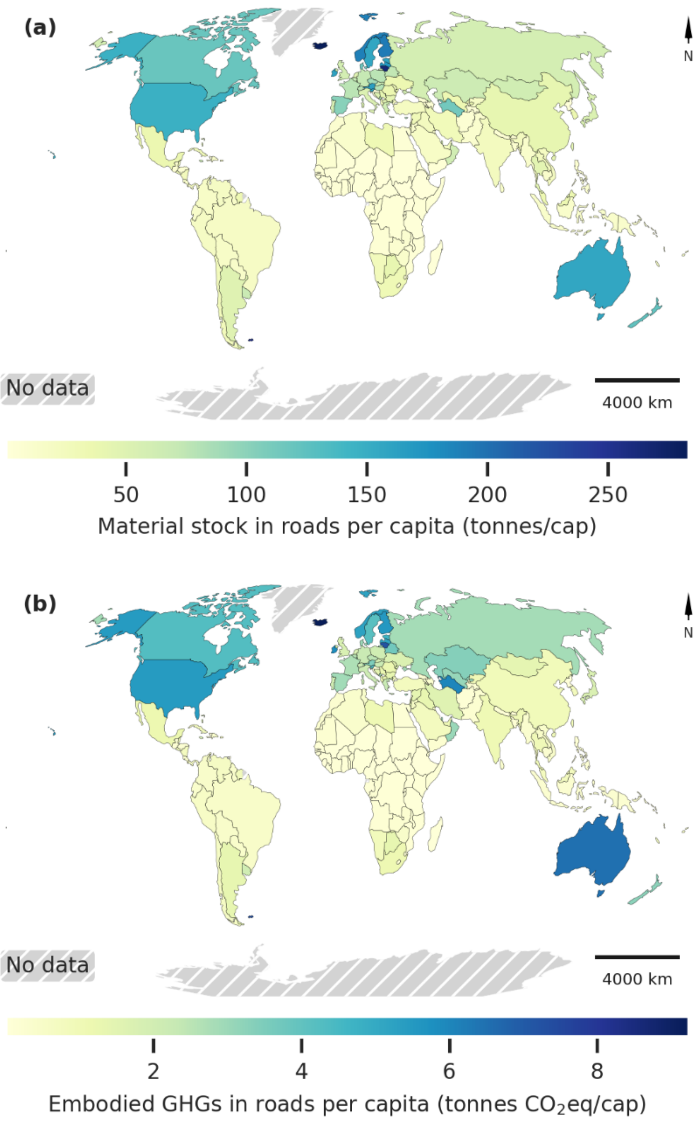

Figure S5 - On top, Figure (a) represents the per capita material stock (tonnes/capita) in the road network per country. On the bottom, Figure (b) represents embodied GHGs (tonnes CO_2-eq/capita) in the road network per country. Material stock and embodied GHGs are based on the combination of GRIP dataset with CIA World Factbook[86] and other statistics[87–94]. Population per country for year 2015 were retrieved from the World Bank[95] and the United Nations[96]

While on the national basis, the United States, China, and India have the largest road material stock. At the per-capita basis, whereas the United States (147 t/cap) is among the countries with the highest material stock per capita, China (42 t/cap) and India (24 t/cap) present much lower values.

S5.3. Further analysis of United States, China, and India

China, the United States, and India stand out with their road material stock and associated GHG emissions (Figure S6).

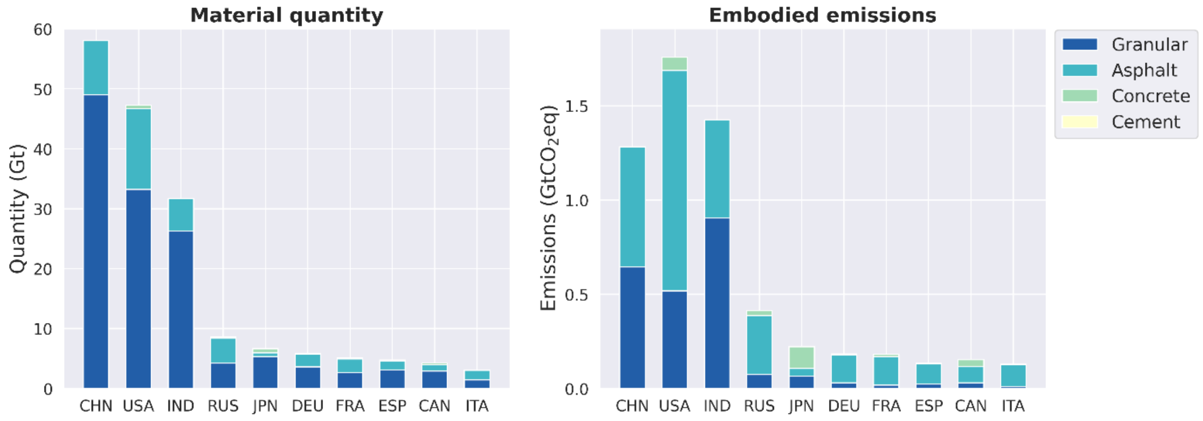

Figure S6 – Top 10 countries with largest road material stock

More specifically, China, the United States, and India (presented in Table S18) are responsible for more than half of the road paved area (52%), material use (54%) and GHG emissions (53%) resulting from construction of the global road network while these three countries only represent 17% of the global land area[97] and were home for 41% of the world population in 2015[95].

Table S18 - Paved area (km^2), material stock (Gt) and embodied GHGs ($GtCO_2$-eq) for the top three countries with largest paved road stock (China, United States, and India)

Country	Paved area		Material stock		Embodied GHGs	
	(10^3 km^2)	Percent of global total	(Gt)	Percent of global total	($GtCO_2$-eq)	Percent of global total
China	46	21.6%	58.1	22.8%	1.3	15.2%
United States	39	18.1%	47.3	18.6%	1.8	20.9%
India	26	12.3%	31.7	12.5%	1.4	17.0%

What stands out in Table S18 is that embodied GHG emissions from roads in China are lower than those in the United States and in India.

An inspection of the material quantity by material type (granular, asphalt, concrete, and cement) in Figure S6 reveals that the ratio asphalt/granular is around 0.2 in China and in India while the ratio (asphalt+concrete)/granular is around 0.4 in the United States. This difference comes from the material intensities defined in the mid-values archetypes: China's ratios asphalt/granular in the archetypes are ranging between 0.1 and 0.3, India's ratios are ranging between 0.2 and 0.3, while for the US, the ratios (asphalt+concrete)/aggregates are ranging between 0.4 and 0.5. However, this finding should be treated cautiously as material intensities of road materials for China are retrieved from one scientific article[22] and uncertainty has been inferred on India's material intensities. The surprisingly lower amount of embodied GHG emissions in China can also be explained by China having lower GHG intensity for both asphalt (Figure S2) and aggregates than the US and India (GHG intensity for aggregates in China is about 10% lower than for the US and 60% lower than for India). We also observe significant differences in the materials' contribution to the GHG emissions in each country. While for both the US and China, GHG emissions from asphalt production are about 5.6–5.8 times the ones from aggregates production, India exhibits a rather high GHG intensity in aggregates production and GHGs from asphalt production are only 2.8 times the ones of aggregates. This high value is due to road transport from the Ecoinvent v3.6[56] process "market for gravel, crushed, IN" used to model GHG emissions from aggregates production.

S6. Roads in urban areas

By 2050, the United Nations projects that 68% of the worldwide population will live in urban areas[98], and the total material stock in cities will reach 90 billion tonnes by 2050 up from 40 billion tonnes in 2010[99]. These numbers illustrate the need for mitigation strategies in material use for the built environment.

Urban areas in our analysis are polygons from NASA Socioeconomic Data and Applications Center[100] and they are defined by nighttime lights and settlements points with buffers.

S6.1. Population and GDP per urban area

In the urban area dataset[100], the attribute population is for the year 2000. We decide to update the population living in each urban area using a population raster file for year 2015 from WorldPop[101]. The raster file is transformed into points, and it is then spatially joined with the urban areas' polygons[100].

The Gross Domestic Product (GDP) for each urban area is estimated using the gridded global dataset for GDP generated by Kummu et al.[102]. This dataset provides GDP per capita at a national or subnational scale (when data available) for years 1990-2015. The average GDP per capita for year 2015 for each urban area is calculated based on a spatial join between the GDP per capita dataset and the urban areas' polygons[100].

S6.2. Urban areas included in the analysis

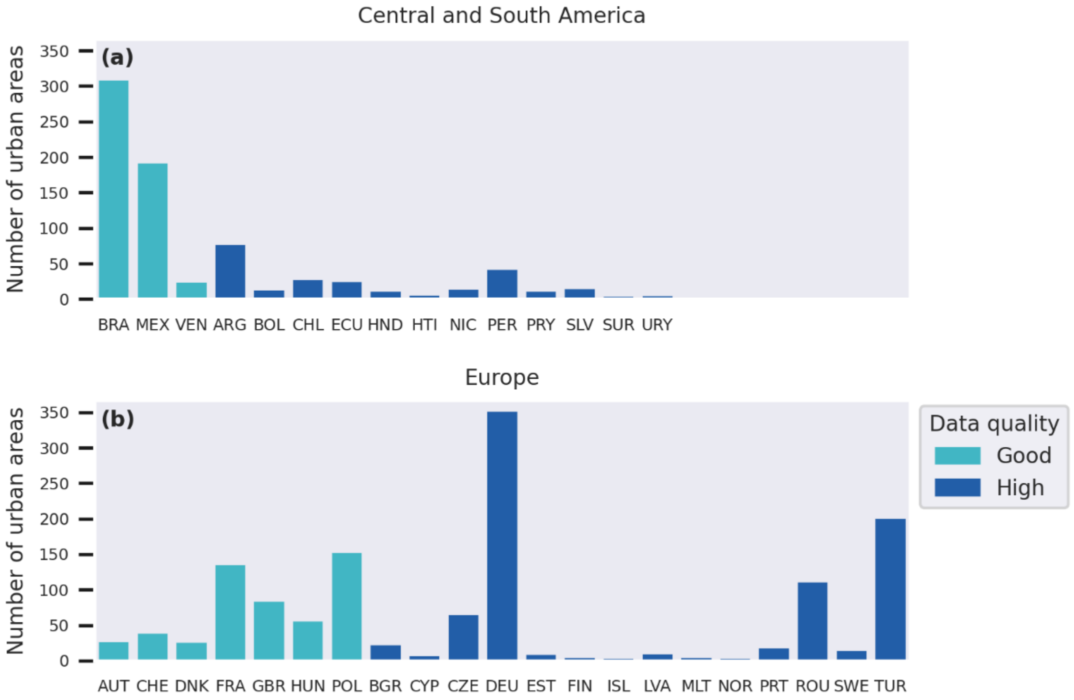

Figure S7 - Number of urban areas located in "good data quality" and "high data quality" countries in (a) Central and South America and (b) Europe

S6.3. Regression models

The urban areas datasets of urban areas in Central and South America and Europe, as described in the manuscript, are both divided into two subsets: train and test datasets. The train datasets are used to get first estimation of OLS regression coefficients and R-squared. The resulting regression models are then applied to the test sets to calculate the Mean Squared Errors. The complete regression analysis (train datasets, test datasets, OLS regression results with train datasets, and OLS regression results with whole datasets) are shown in the Excel document. The train and test datasets in the Excel document are one sample. Several train and test datasets were generated, the p-values for each variable in the resulting regression models were retrieved to calculate the median p-values and determining if the variables were statistically significant.

Pearson Correlation Coefficients between the variables are shown in Figure S8. These coefficients provide an indication of the relationship between road material stock and the three variables considered (density, GDP per capita, and urban areas).

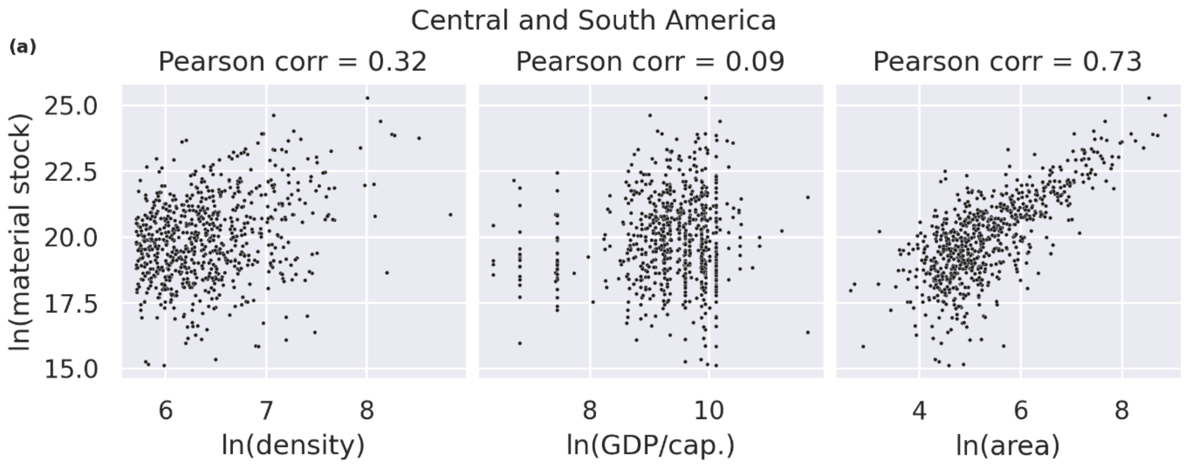

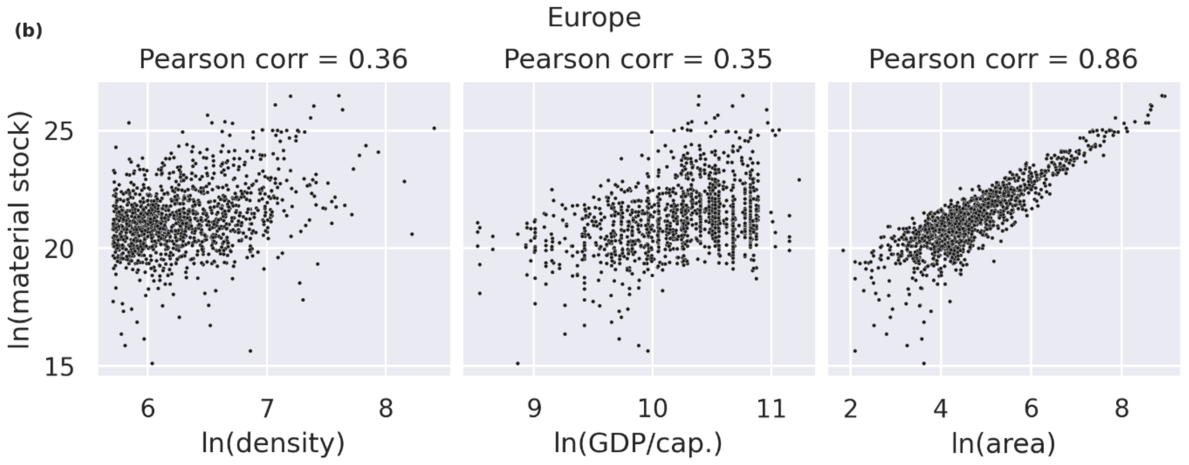

Figure S8 – Pearson Correlation Coefficients between variables for urban areas (a) Central and South America and (b) in Europe

Graphs of Observations-Predictions for the models in Table 2 are presented in Figure S9. We observe a tail of data points on the left part of the graphs for which the prediction is larger than the observation. We introduce an indicator: the percentage of each urban area's total surface area covered by paved road surface, calculated as the paved road surface area divided by the urban area. Urban areas having less than 0.25% of their area covered by paved road surface are identified in red in Figure S9.

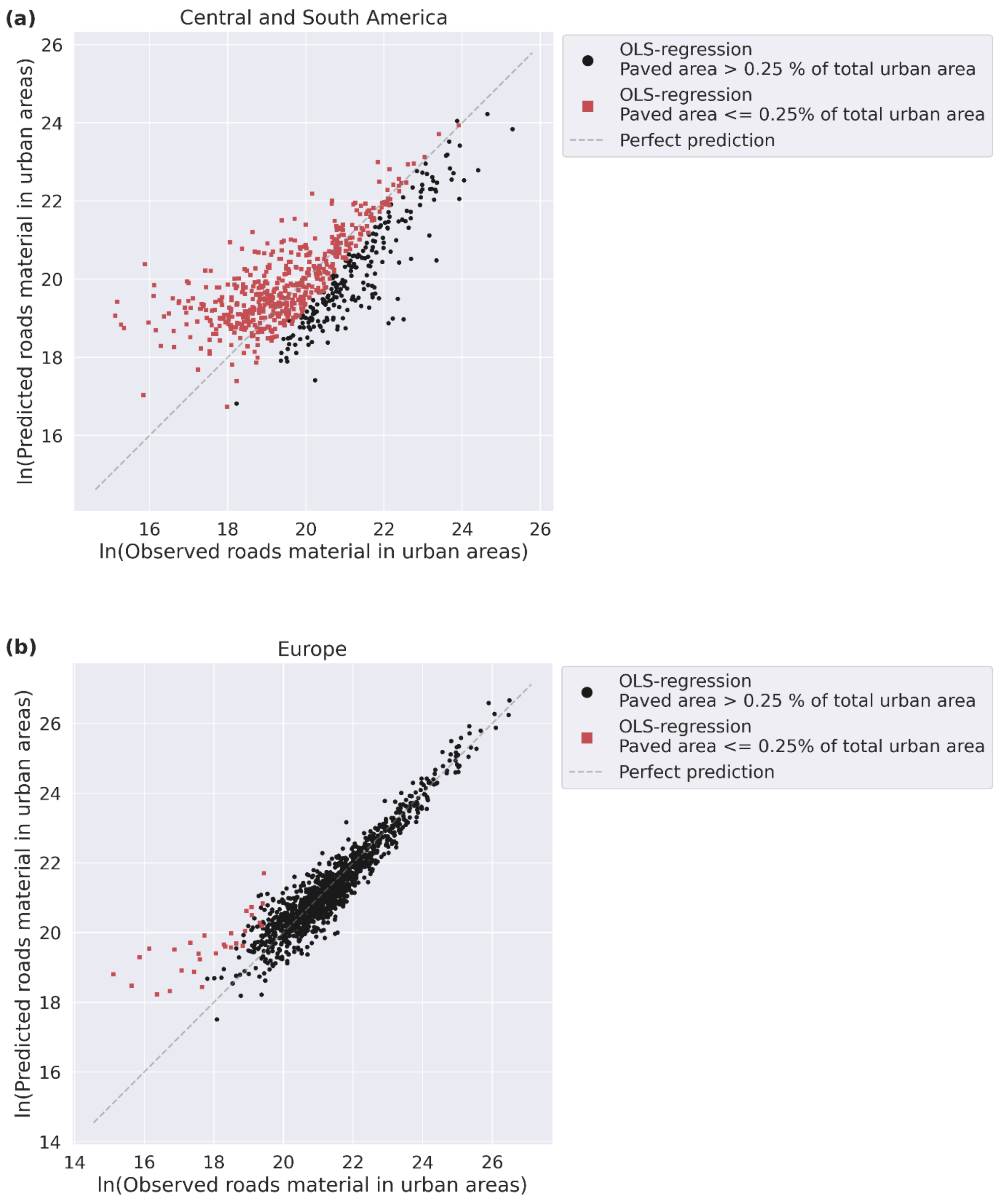

Figure S9 – Graphs of Observations-Predictions of log10 of road material stock for all the urban areas resulting from the OLS regression model: (a) Central and South America, (b) Europe. In blue, identification of urban areas with paved area ≤ 0.25% of total urban area.

Two hypotheses are raised to explain this observation: (1) the tail could come from the limitations of the GRIP dataset leading to an underestimation of the material stock in many urban areas, (2) the model is biased towards urban areas with higher material stocks and does not adequately capture other indicators that might explain the lower than predicted values for urban areas in the left tail. Further

research would be needed to test these hypotheses and improve the model (for example including parameters such as land use mix, vehicle ownership, public transportation use). Other more difficult to model parameters—outside the scope of this paper—are also likely to influence road material stock, for example cultural preferences, government policy and priorities, and urban planning practices.

S7. National and regional studies from the literature

In addition to comparing our global road material stock estimate with buildings material stock (in the manuscript), we can compare it with prior bottom-up material stock studies on other transport infrastructure. Mao et al.[103] estimated 2460 Mt of materials in the subway networks of over 200 cities in 2020 which is about 1% of the global road material stock we estimated.

S7.1. Material stock per capita

We compare our results (road material stock per capita) with the available national and regional studies from the literature.

Table S19 – Material stock per capita compared to the literature

Country/region	Year	Material stock (tonnes) per capita	Source
Canada (Kitchener and Waterloo)	2018	72	Mollaei et al.[104]
Canada	-	119	This study
United States	2015	47	Miatto et al.[105]
United States	-	147	This study
EU25	2009	85	Wiedenhofer et al.[106]
Western Europe*	-	78	This study
Europe (GRIP)	-	69	This study
Germany	2009	67	Wiedenhofer et al.[106]
Germany	2018	141	Haberl et al.[107]
Germany	-	71	This study
Austria	2018	233	Haberl et al.[107]
Austria	-	164	This study
Norway	2017	80	Ebrahimi et al.[108]
Norway	-	189	This study
United Kingdom (Salford Quays)	2004	23	Tanikawa et al.[43]
United Kingdom	-	59	This study
Japan (Wakayama City centre)	2004	27	Tanikawa et al.[43]
Japan	2010	29 (GIS data)<br>45 (Prefecture-level data)	Tanikawa et al.[109]
Japan	-	52	This study
China (Beijing – Inside 5th ring)	2011	12	Guo et al.[110]
China (Shandong Peninsula)	2013	59	Guo et al.[111]

China	-	42	This study
Vietnam	2012	30	Nguyen et al.[25]
Vietnam	-	21	This study

* Austria, Belgium, Denmark, Finland, France, Germany, Greece, Iceland, Ireland, Italy, Luxembourg, Netherlands, Norway, Portugal, Spain, Sweden, Switzerland, United Kingdom

S7.2. Roads-to-Buildings ratios

We calculate Roads-to-Residential Buildings ratio (RtRB) and Roads-to-Buildings ratio (RtB) for a few individual countries for which material stock in buildings was available in the literature using the ODYM-RECC model[112] and the results from Deetman et al.[113].

Table S20 – Roads-to-Residential Buildings ratio (RtRB) and Roads-to-Buildings ratio (RtB) based on Material Stock (MS) of paved roads (this study) and Material Stock (MS) of buildings from existing literature

Country	MS [Gt] paved roads (this study)	Residential Buildings (ODYM-RECC[112])		Residential Buildings (Deetman et al.[113])		All Buildings (Deetman et al.[113])	
		MS [Gt]	RtRB	MS [Gt]	RtRB	MS [Gt]	RtB
Canada	4.2	1.3	3.3	1.9	2.3	2.5	1.7
China	58.1	39.7	1.5	128.5	0.5	134.1	0.4
United States	47.3	12.8	3.7	10.0	4.7	17.2	2.7
India	31.7	9.3	3.4	15.1	2.1	17.6	1.8
Japan	6.6	5.9	1.1	3.1	2.1	5.4	1.2
Germany	5.8	3.5	1.7	-	-	-	-
France	5.1	2.6	1.9	-	-	-	-
Spain	4.7	1.9	2.5	-	-	-	-
United Kingdom	3.9	2.5	1.6	-	-	-	-
Italy	3.1	2.4	1.3	-	-	-	-
Poland	2.9	1.4	2.1	-	-	-	-
Western Europe**	32.8	-	-	20.6	1.6	28.0	1.2

* (after length re-estimation)

** Paved roads (this study): Austria, Belgium, Denmark, Finland, France, Germany, Greece, Iceland, Ireland, Italy, Luxembourg, Netherlands, Norway, Portugal, Spain, Sweden, Switzerland, United Kingdom

Buildings[113]: Andorra, Austria, Belgium, Denmark, Faeroe Islands, Finland, France, Germany, Gibraltar, Greece, Iceland, Ireland, Italy, Liechtenstein, Luxembourg, Malta, Monaco, Netherlands, Norway, Portugal, San Marino, Spain, Sweden, Switzerland, United Kingdom, Vatican City State

References

(1) Meijer, J. R.; Huijbegts, M. A. J.; Schotten, C. G. J.; Schipper, A. M. Global Patterns of Current and Future Road Infrastructure. *Environ. Res. Lett.* **2018**, *13* (6), 064006. https://doi.org/10.1088/1748-9326/aabd42.

(2) Schipper, A. M.; Hilbers, J. P.; Meijer, J. R.; Antão, L. H.; Benítez-López, A.; de Jonge, M. M. J.; Leemans, L. H.; Scheper, E.; Alkemade, R.; Doelman, J. C.; Mylius, S.; Stehfest, E.; van Vuuren, D. P.; van Zeist, W.-J.; Huijbregts, M. A. J. Projecting Terrestrial Biodiversity Intactness with GLOBIO 4. *Glob. Chang. Biol.* **2020**, *26* (2), 760–771. https://doi.org/10.1111/gcb.14848.

(3) Dou, X.; Wang, Y.; Ciais, P.; Chevallier, F.; Davis, S. J.; Crippa, M.; Janssens-Maenhout, G.; Guizzardi, D.; Solazzo, E.; Yan, F.; Huo, D.; Zheng, B.; Zhu, B.; Cui, D.; Ke, P.; Sun, T.; Wang, H.; Zhang, Q.; Gentine, P.; Deng, Z.; Liu, Z. Near-Real-Time Global Gridded Daily CO2 Emissions. *The Innovation* **2022**, *3* (1), 100182. https://doi.org/10.1016/j.xinn.2021.100182.

(4) Beck, H. E.; Zimmermann, N. E.; McVicar, T. R.; Vergopolan, N.; Berg, A.; Wood, E. F. Present and Future Köppen-Geiger Climate Classification Maps at 1-Km Resolution. *Sci. Data* **2018**, *5* (1), 180214. https://doi.org/10.1038/sdata.2018.214.

(5) Jordahl, K.; Bossche, J. V. den; Fleischmann, M.; Wasserman, J.; McBride, J.; Gerard, J.; Tratner, J.; Perry, M.; Badaracco, A. G.; Farmer, C.; Hjelle, G. A.; Snow, A. D.; Cochran, M.; Gillies, S.; Culbertson, L.; Bartos, M.; Eubank, N.; maxalbert; Bilogur, A.; Rey, S.; Ren, C.; Arribas-Bel, D.; Wasser, L.; Wolf, L. J.; Journois, M.; Wilson, J.; Greenhall, A.; Holdgraf, C.; Filipe; Leblanc, F. *Geopandas/Geopandas: V0.9.0*; Zenodo, 2021. https://doi.org/10.5281/zenodo.4569086.

(6) Gillies, S.; others. *Rasterio: Geospatial Raster I/O for Python Programmers*; Mapbox, 2013.

(7) Virtanen, P.; Gommers, R.; Oliphant, T. E.; Haberland, M.; Reddy, T.; Cournapeau, D.; Burovski, E.; Peterson, P.; Weckesser, W.; Bright, J.; van der Walt, S. J.; Brett, M.; Wilson, J.; Millman, K. J.; Mayorov, N.; Nelson, A. R. J.; Jones, E.; Kern, R.; Larson, E.; Carey, C. J.; Polat, İ.; Feng, Y.; Moore, E. W.; VanderPlas, J.; Laxalde, D.; Perktold, J.; Cimrman, R.; Henriksen, I.; Quintero, E. A.; Harris, C. R.; Archibald, A. M.; Ribeiro, A. H.; Pedregosa, F.; van Mulbregt, P.; SciPy 1.0 Contributors. SciPy 1.0: Fundamental Algorithms for Scientific Computing in Python. *Nat. Methods* **2020**, *17*, 261–272. https://doi.org/10.1038/s41592-019-0686-2.

(8) Charles W. Schwartz; Gary E. Elkins; Rui Li; Beth A. Visintine; Barton Barton; Barton Forman; Gonzalo R. Rada; Jonathan L. Groeger. *Evaluation of LTPP Climatic Data for Use in Mechanistic-Empirical Pavement Design Guide Calibration and Other Pavement Analysis*; Federal Highway Administration, 2015.

(9) Tenkanen, H.; Pyrosm contributors. *HTenkanen/Pyrosm: V.0.6.0*; 2020.

(10) Federal Highway Administration. *Highway Functional Classification Concepts, Criteria and Procedures*. Planning Processes - Statewide Transportation Planning. https://www.fhwa.dot.gov/planning/processes/statewide/related/highway_functional_classifications/section03.cfm (accessed 2022-03-14).

(11) Transportation Association of Canada (TAC). *Geometric Design Guide for Canadian Roads*; 2017.

(12) Ministerio de Vivienda y Urbanismo [Chilean Ministry of housing and urbanism]. *Manual de Vialidad Urbana - Recomendaciones Para El Diseño de Elementos de Infraestructura Vial Urbana [Manual of Urban Roads - Recommendations for the Design of Elements of Urban Road Infrastructure]*; 2009.

(13) CSIR Building and Construction Technology. Chapter 7 - Roads: Geometric Design and Layout Planning. In *Human Settlement Planning and Design*; 2005; Vol. 2.

(14) European Road Safety Observatory. *Road Safety Syntheses: Motorways 2018*; 2018; p 36.

(15) Teodorović, D.; Janić, M. Transportation, Environment, and Society. In *Transportation Engineering: Theory, Practice and Modeling*; Butterworth-Heinemann, 2017; pp 719–858. https://doi.org/10.1016/B978-0-12-803818-5.00011-1.

(16) Ministerio de Transportes Movilidad y Agenda Urbana [Spanish Ministry of Transport Mobility and Urban Agenda]. *Norma 3.1 IC - Trazado [Roadway Geometric Design Standards]*; 2016.

(17) Highways England. *Design Manual for Roads and Bridges - CD 226: Design for New Pavement Construction*; 2020.

(18) UK Department of Transport. *Manual for Streets*; ISBN: 978-0-7277-3501-0; 2007.

(19) German Road and Transportation Research Association (FGSV). *Guidelines for the Design of Motorways, RAA, Edition 2008, Translation 2011*; 2011.

(20) German Road and Transportation Research Association (FGSV). *Directives for the Design of Urban Roads, RASt 06, Edition 2006, Translation 2012*; 2012.

(21) Alzard, M. H.; Maraqa, M. A.; Chowdhury, R.; Khan, Q.; Albuquerque, F. D. B.; Mauga, T. I.; Aljunadi, K. N. Estimation of Greenhouse Gas Emissions Produced by Road Projects in Abu Dhabi, United Arab Emirates. *Sustainability* **2019**, *11* (8). https://doi.org/10.3390/su11082367.

(22) Yu, B.; Li, L.; Tian, X.; Yu, Q.; Liu, J.; Wang, Q. Material Stock Quantification and Environmental Impact Analysis of Urban Road Systems. *Transp. Res. D Trans. Environ* **2021**, *93*, 102756. https://doi.org/10.1016/j.trd.2021.102756.

(23) Road Bureau. *Roads in Japan*; Ministry of Land, Infrastructure, Transport and Tourism, 2018.

(24) Indian Roads Congress. *Geometric Design Standards for Urban Roads and Streets - IRC:86-2018*; 2018.

(25) Nguyen, T. C.; Fishman, T.; Miatto, A.; Tanikawa, H. Estimating the Material Stock of Roads: The Vietnamese Case Study. *J. Ind. Ecol.* **2019**, *23* (3), 663–673. https://doi.org/10.1111/jiec.12773.

(26) Austroads. *Guide to Road Design - Part 3: Geometric Design*; 2021.

(27) Gregory, J.; AzariJafari, H.; Vahidi, E.; Guo, F.; Ulm, F.-J.; Kirchain, R. The Role of Concrete in Life Cycle Greenhouse Gas Emissions of US Buildings and Pavements. *Proc. Natl. Acad. Sci* **2021**, *118* (37), e2021936118. https://doi.org/10.1073/pnas.2021936118.

(28) Federal Highway Administration. *LTPP InfoPave, Research quality pavement performance information*. http://www.infopave.com/.

(29) British Columbia Ministry of Transportation and Infrastructure. *Pavement Structure Design Guidelines, Technical Circular T-01/15*; 2015; pp 1–20.

(30) City of Toronto. Pavement Structural Design Matrix—Minimum Requirements, 2019.

(31) Ministry of Transportation of Ontario. *Pavement Design and Rehabilitation Manual*; Ministry of Transportation of Ontario, 2013.

(32) Thurber Engineering Ltd. *Equivalent Pavement Designs for Municipalities: Rigid and Flexible Pavements, Province of British Columbia*; Report to Cement Association of Canada, 2016.

(33) U.S. Federal Highway Administration. Long-Term Pavement Performance (LTPP) Program. *InfoPave Data*, 2021.

(34) Subdirección de Pavimentación y Obras Viales de Chile [Subdirectorate of Paving and Road Works of Chile]. Cap. N°1: Diseño Estructural de Pavimentos [Chap. N°1: Structural Pavement

Design]. In *Manual de Obras de Vialidad, Pavimentación y Aguas Lluvias, versión 2020*; 2020.

(35) Dirección General de Servicios Técnicos [General Directorate of Technical Services]. *Catálogo de Secciones Estructurales de Pavimentos Para Las Carreteras de La República Mexicana [Catalog of Structural Sections of Pavements for Highways in the Mexican Republic]*.

(36) Department of Transport, Republic of South Africa. *Technical Recommendations for Highways Draft TRH4 - Structural Design of Flexbible Pavements for Interurban and Rural Roads*; 1996.

(37) The South African National Roads Agency. *Technical Recommendations for Highways TRH3 - Design and Construction of Surfacing Seals*; Republic of South Africa, 2007.

(38) German Road and Transportation Research Association (FGSV). *Guidelines for the Standardisation of Pavement Structures of Traffic Areas, RStO 12, Edition 2012, Translation 2015*; 978-3-86446-021–0; 2015; pp 56–56.

(39) Forschungsgesellschaft Straße - Schiene - Verkehr (FSV) [Austrian Road-Rail-Transport Research Society]. *RVS 03.08.63 Oberbaubemessung [Pavement Superstructure Dimensioning]*; 2016.

(40) Ministerio de Transportes Movilidad y Agenda Urbana [Spanish Ministry of Transport Mobility and Urban Agenda]. *Norma 6.1 IC - Secciones de Firme [Pavement Cross Sections]*; 2003.

(41) Consiglio Nazionale delle Ricerche (CNR) [Italian National Research Council]. Catalogo Delle Pavimentazioni Stradali [Road Pavement Catalogue]. *Bollettino Ufficiale del CNR Parte IV - Norme tecniche n. 178* **1995**, *15/9/1995*.

(42) Department of Municipalities and Transport, United Arab Emirates. *Pavement Design Manual - Document TR-513*, Second.; 2021.

(43) Tanikawa, H.; Hashimoto, S. Urban Stock over Time: Spatial Material Stock Analysis Using 4d-GIS. *Build. Res. Inf.* **2009**, *37* (5–6), 483–502. https://doi.org/10.1080/09613210903169394.

(44) Indian Roads Congress. *Guidelines for the Design of Flexible Pavements - IRC:37-2018*, Fourth Revision.; 2018.

(45) Miatto, A.; Dawson, D.; Dac, P.; Kanaoka, K. S.; Tanikawa, H. The Urbanisation-Environment Conflict: Insights from Material Stock and Productivity of Transport Infrastructure in Hanoi, Vietnam. *J. Environ. Manage.* **2021**, *294* (113007). https://doi.org/10.1016/j.jenvman.2021.113007.

(46) Austroads. *Guide to Pavement Technology - Part 2: Pavement Structural Design*; 2008.

(47) Waka Kotahi NZ Transport Agency. *NZTA M10 Notes: 2020 - Notes to the Specification for Dense Graded Asphaltic Concrete*; 2020.

(48) Athena Sustainable Materials Institute. *Athena Pavement LCA*; 2021.

(49) Visser, A. T. Potential of South African Road Technology for Application in China. *J. Traffic Transp. Eng. (Engl. Ed.)* **2017**, *4* (2), 113–117. https://doi.org/10.1016/j.jtte.2017.03.004.

(50) Federal Highway Administration. *Highway Statistics 2017*. https://www.fhwa.dot.gov/policyinformation/statistics/2017/.

(51) EAPA; NAPA. *The Asphalt Paving Industry: A Global Perspective*; Brussels, Belgium, 2011.

(52) Hall, K.; Dawood, D.; Vanikar, S.; Tally, R., Jr.; Cackler, T.; Correa, A.; Deem, P.; Duit, J.; Geary, G.; Gisi, A.; Hanna, A.; Kosmatka, S.; Rasmussen, R.; Tayabji, S.; Voigt, G. *Long-Life Concrete Pavements in Europe and Canada (No. FHWA-PL-07-027)*; 2007.

(53) Sutherland, J.; Humm, D.; Chrimes, M. Historic Concrete: The Background to Appraisal. *Historic Concrete: The Background to Appraisal*, 2001.

(54) Kubo, K. *Pavement Maintenance in Japan*. Road Conference 2017 - International Symposium. https://road.or.jp/international/pdf/32_AM6.pdf.

(55) Mistry, R.; Roy, T. K. Effect of Using Fly Ash as Alternative Filler in Hot Mix Asphalt. *Perspectives in Science* **2016**, *8*, 307–309. https://doi.org/10.1016/j.pisc.2016.04.061.

(56) Wernet, G.; Bauer, C.; Steubing, B.; Reinhard, J.; Moreno-Ruiz, E.; Weidema, B. The Ecoinvent Database Version 3 (Part I): Overview and Methodology. *Int. J. Life Cycle Assess.* **2016**, *21* (9), 1218–1230. https://doi.org/10.1007/s11367-016-1087-8.

(57) Masnadi, M. S.; El-Houjeiri, H. M.; Schunack, D.; Li, Y.; Englander, J. G.; Badahdah, A.; Monfort, J.-C.; Anderson, J. E.; Wallington, T. J.; Bergerson, J. A.; Gordon, D.; Koomey, J.; Przesmitzki, S.; Azevedo, I. L.; Bi, X. T.; Duffy, J. E.; Heath, G. A.; Keoleian, G. A.; McGlade, C.; Meehan, D. N.; Yeh, S.; You, F.; Wang, M.; Brandt, A. R. Global Carbon Intensity of Crude Oil Production. *Science* **2018**, *361* (6405), 851–853. https://doi.org/10.1126/science.aar6859.

(58) Wildnauer, M.; Mulholland, E.; Liddie, J. *Life Cycle Assessment of Asphalt Binder*; 2019.

(59) Eurobitume. *The Eurobitume Life-Cycle Inventory for Bitumen, Version 3.1*; European Bitumen Association: Brussels, Belgium, 2020.

(60) U.S. Energy Information Administration. *Global crude oil supply disruptions and strong demand support high oil prices*. https://www.eia.gov/todayinenergy/detail.php?id=12891 (accessed 2022-03-11).

(61) U.S. Energy Information Administration. *Oil and petroleum products explained*. https://www.eia.gov/energyexplained/oil-and-petroleum-products/imports-and-exports.php (accessed 2021-01-06).

(62) U.S. Energy Information Administration. *How much of the crude oil produced in the United States is consumed in the United States?*. https://www.eia.gov/tools/faqs/faq.php?id=268&t=6 (accessed 2022-01-06).

(63) IEA. *World energy statistics*. IEA World Energy Statistics and Balances. https://doi.org/10.1787/enestats-data-en (accessed 2021-06-10).

(64) CEPII. *BACI (Base pour l'Analyse du Commerce International) [World Database of International Trade at the Product Level]*. http://www.cepii.fr/CEPII/en/bdd_modele/bdd_modele.asp (accessed 2021-05-11).

(65) Gaulier, G.; Zignago, S. *BACI: International Trade Database at the Product-Level - The 1994-2007 Version*; 2010.

(66) Jing, L.; El-Houjeiri, H. M.; Monfort, J.-C.; Brandt, A. R.; Masnadi, M. S.; Gordon, D.; Bergerson, J. A. Carbon Intensity of Global Crude Oil Refining and Mitigation Potential. *Nat. Clim. Chang.* **2020**, *10* (6), 526–532. https://doi.org/10.1038/s41558-020-0775-3.

(67) Majeau-Bettez, G.; Strømman, A. H. Documentation for Arda Calculator. 2016.

(68) EAPA. *Environmental Guidelines on Best Available Techniques (BAT) for the Production of Asphalt Paving Mixes*; Brussels, Belgium, 2007.

(69) Kristjánsdóttir, Ó.; Muench, S. T.; Michael, L.; Burke, G. Assessing Potential for Warm-Mix Asphalt Technology Adoption. *Transp Res Rec.* **2007**, *2040* (1), 91–99. https://doi.org/10.3141/2040-10.

(70) Myers, R.; Shrager, B.; Klamm, S.; Marinshaw, R.; Marshall, A. *Hot Mix Asphalt Plants - Emission Assessment Report*; U.S. Environmental Protection Agency, 2000.

(71) Vidal, R.; Moliner, E.; Martínez, G.; Rubio, M. C. Life Cycle Assessment of Hot Mix Asphalt and Zeolite-Based Warm Mix Asphalt with Reclaimed Asphalt Pavement. *Resour. Conserv. Recycl.* **2013**, *74*, 101–114. https://doi.org/10.1016/j.resconrec.2013.02.018.

(72) Stripple, H. *Life Cycle Assessment of Road - A Pilot Study for Inventory Analysis - Second Edition*; IVL Swedish Environmental Research Institute: Gothenburg, Sweden, 2001.

(73) Franzitta, V.; Longo, S.; Sollazzo, G.; Cellura, M.; Celauro, C. Primary Data Collection and Environmental/Energy Audit of Hot Mix Asphalt Production. *Energies* **2020**, *13* (8). https://doi.org/10.3390/en13082045.

(74) Ang, B. W.; Fwa, T. F.; Ng, T. T. Analysis of Process Energy Use of Asphalt-Mixing Plants. *Energy* **1993**, *18* (7), 769–777. https://doi.org/10.1016/0360-5442(93)90035-C.

(75) Tahmoorian, F.; Bracken, R.; Wheatley, M.; Yeaman, J. Life Cycle Assessment of Hot Mix Asphalt Containing Recycled Materials: Case Study in Australia. In *Airfield and Highway Pavements 2019*; 2019; pp 143–149. https://doi.org/10.1061/9780784482476.016.

(76) Vega A., D. L.; Santos, J.; Martinez-Arguelles, G. Life Cycle Assessment of Hot Mix Asphalt with Recycled Concrete Aggregates for Road Pavements Construction. *Int. J. Pavement Eng.* **2020**, *0* (0), 1–14. https://doi.org/10.1080/10298436.2020.1778694.

(77) Thenoux, G.; González, Á.; Dowling, R. Energy Consumption Comparison for Different Asphalt Pavements Rehabilitation Techniques Used in Chile. *Resour. Conserv. Recycl.* **2007**, *49* (4), 325–339. https://doi.org/10.1016/j.resconrec.2006.02.005.

(78) Hasan, U.; Whyte, A.; Al Jassmi, H. Life Cycle Assessment of Roadworks in United Arab Emirates: Recycled Construction Waste, Reclaimed Asphalt Pavement, Warm-Mix Asphalt and Blast Furnace Slag Use against Traditional Approach. *J. Clean. Prod.* **2020**, *257*, 120531. https://doi.org/10.1016/j.jclepro.2020.120531.

(79) Giani, M. I.; Dotelli, G.; Brandini, N.; Zampori, L. Comparative Life Cycle Assessment of Asphalt Pavements Using Reclaimed Asphalt, Warm Mix Technology and Cold in-Place Recycling. *Resour. Conserv. Recycl.* **2015**, *104*, 224–238. https://doi.org/10.1016/j.resconrec.2015.08.006.

(80) Siverio Lima, M. S.; Hajibabaei, M.; Hesarkazzazi, S.; Sitzenfrei, R.; Buttgereit, A.; Queiroz, C.; Tautschnig, A.; Gschösser, F. Environmental Potentials of Asphalt Materials Applied to Urban Roads: Case Study of the City of Münster. *Sustainability* **2020**, *12* (15). https://doi.org/10.3390/su12156113.

(81) Ma, F.; Sha, A.; Lin, R.; Huang, Y.; Wang, C. Greenhouse Gas Emissions from Asphalt Pavement Construction: A Case Study in China. *Int. J. Environ. Res. Public Health* **2016**, *13* (3). https://doi.org/10.3390/ijerph13030351.

(82) Telle, R.; Hoven, B. *Båttransport av asfalt - Varige veger 2011-2015 [Boat transport of asphalt - Durable roads 2011-2015]*; Statens vegvesen, 2016.

(83) Grael, P. F. F.; Oliveira, L. S. B. L.; Oliveira, D. S. B. L.; Bezerra, B. S. Life Cycle Inventory and Impact Assessment for an Asphalt Pavement Road Construction—a Case Study in Brazil. *Int. J. Life Cycle Assess.* **2021**, *26* (2), 402–416. https://doi.org/10.1007/s11367-020-01842-5.

(84) Mao, R.; Duan, H.; Dong, D.; Zuo, J.; Song, Q.; Liu, G.; Hu, M.; Zhu, J.; Dong, B. Quantification of Carbon Footprint of Urban Roads via Life Cycle Assessment: Case Study of a Megacity-Shenzhen, China. *J. Clean. Prod.* **2017**, *166*, 40–48. https://doi.org/10.1016/j.jclepro.2017.07.173.

(85) Biswas, W. K. Carbon Footprint and Embodied Energy Assessment of a Civil Works Program in a Residential Estate of Western Australia. *Int. J. Life Cycle Assess.* **2014**, *19* (4), 732–744. https://doi.org/10.1007/s11367-013-0681-2.

(86) Central Intelligence Agency. *The World Factbook - Roadways*. The World Factbook. https://www.cia.gov/the-world-factbook/field/roadways/ (accessed 2021-10-19).

(87) Statistisk Sentralbyrå [Statistics Norway]. *Samferdsel i kommuner og fylkeskommuner - 11845: Veier, parkering, belysning, holdeplasser, etter region, statistikkvariabel og år [Transport in municipalities and county municipalities - 11845: Roads, parking spaces, road lights, bus stops, by region, statistical variable and year]*. https://www.ssb.no/statbank/sq/10061434 (accessed 2022-01-03).

(88) Tilastokeskus [Statistics Finland]. *Pavements by Pavement, Year and Information*. https://pxnet2.stat.fi:443/PXWeb/sq/506118b7-d00a-4e51-953b-755bbb77829e (accessed 2022-01-03).

(89) Bureau of Infrastructure and Transport Research Economics (BITRE). *Yearbook 2020: Australian Infrastructure Statistics*; Statistical Report; BITRE: Canberra ACT, 2020.

(90) Ministry of Road Transport and Highways. *Basic Road Statistics of India 2016-2017*; Government of India, 2017.

(91) Brüning Maldonado, W. *Dirección de Vialidad: Infrastructura Vial en Chile [Directorate of Roads: Road Infrastructure in Chile]*. http://icha.cl/wp-content/uploads/2016/08/1-Presentaci%C3%B3n-de-Walter-Br%C3%BCning-Director-Nacional-de-Vialidad.pdf (accessed 2022-01-03).

(92) Royaume du Maroc - Ministère de l'Equipement et de l'Eau [Kingdom of Morocco - Ministry of Equipment and Water]. *Infrastructure routières: Importance du réseau [Road infrastructure: Importance of the network]*. http://www.equipement.gov.ma/Infrastructures-Routieres/Reseau-Routier-du-Royaume/Pages/Importance-du-reseau.aspx (accessed 2022-01-03).

(93) Venezolana de Televisión. *Al menos 32 mil kilómetros de vías pavimentadas en el país en 20 años de Revolución [At least 32 thousand kilometers of paved roads in the country in 20 years of Revolution]*. https://www.vtv.gob.ve/vias-pavimentadas-20-anos-revolucion-final/ (accessed 2022-01-03).

(94) Gaviria Muñoz, S. *Efecto Macroeconómicos de las Obras de Infraestructura [Macroeconomic Effect of Infrastructure Works]*. https://colaboracion.dnp.gov.co/CDT/Prensa/Presentaciones/2016-05-18%20Director-Seminario%20Infraestructura%20ANIF.pdf (accessed 2022-01-03).

(95) World Bank. *Population, total (Year 2015)*. World Bank Open Data. https://data.worldbank.org/ (accessed 2022-01-28).

(96) United Nations, Department of Economic and Social Affairs, Population Division. *World Population Prospects: The 2022 Revision*. https://population.un.org/wpp/ (accessed 2022-10-24).

(97) World Bank. *Land area*. World Bank Open Data. https://data.worldbank.org/ (accessed 2022-01-27).

(98) United Nations; Department of Economic and Social Affairs; Population Division. *World Urbanization Prospects 2018: Highlights*; 2019.

(99) Swilling, M.; Hajer, M.; Baynes, T.; Bergesen, J.; Labbé, F.; Musango, J. K.; Ramaswami, A.; Robinson, B.; Salat, S.; Suh, S. *The Weight of Cities: Resource Requirements of Future Urbanization*; IRP Reports, 2018.

(100) Center for International Earth Science Information Network - CIESIN - Columbia University. Global Urban Heat Island (UHI) Data Set, 2013. NASA Socioeconomic Data and Applications Center (SEDAC): Palisades, NY 2016.

(101) WorldPop (www.worldpop.org - School of Geography and Environmental Science, University of Southampton; Department of Geography and Geosciences, University of Louisville; Departement de Geographie, Universite de Namur); Center for International Earth Science

Information Network (CIESIN), Columbia University. Global High Resolution Population Denominators Project. 2018.

(102) Kummu, M.; Taka, M.; Guillaume, J. H. A. Gridded Global Datasets for Gross Domestic Product and Human Development Index over 1990–2015. *Sci. Data* **2018**, *5* (1), 180004. https://doi.org/10.1038/sdata.2018.4.

(103) Mao, R.; Bao, Y.; Duan, H.; Liu, G. Global Urban Subway Development, Construction Material Stocks, and Embodied Carbon Emissions. *Humanit. Soc. Sci. Commun.* **2021**, *8* (1), 83. https://doi.org/10.1057/s41599-021-00757-2.

(104) Mollaei, A.; Ibrahim, N.; Habib, K. Estimating the Construction Material Stocks in Two Canadian Cities: A Case Study of Kitchener and Waterloo. *J. Clean. Prod.* **2021**, *280*, 124501. https://doi.org/10.1016/j.jclepro.2020.124501.

(105) Miatto, A.; Schandl, H.; Wiedenhofer, D.; Krausmann, F.; Tanikawa, H. Modeling Material Flows and Stocks of the Road Network in the United States 1905–2015. *Resour. Conserv. Recycl.* **2017**, *127*, 168–178. https://doi.org/10.1016/j.resconrec.2017.08.024.

(106) Wiedenhofer, D.; Steinberger, J. K.; Eisenmenger, N.; Haas, W. Maintenance and Expansion: Modeling Material Stocks and Flows for Residential Buildings and Transportation Networks in the EU25. *J. Ind. Ecol.* **2015**, *19* (4), 538–551. https://doi.org/10.1111/jiec.12216.

(107) Haberl, H.; Wiedenhofer, D.; Schug, F.; Frantz, D.; Virág, D.; Plutzar, C.; Gruhler, K.; Lederer, J.; Schiller, G.; Fishman, T.; Lanau, M.; Gattringer, A.; Kemper, T.; Liu, G.; Tanikawa, H.; van der Linden, S.; Hostert, P. High-Resolution Maps of Material Stocks in Buildings and Infrastructures in Austria and Germany. *Environ. Sci. Technol.* **2021**, *55* (5), 3368–3379. https://doi.org/10.1021/acs.est.0c05642.

(108) Ebrahimi, B.; Rosado, L.; Wallbaum, H. Machine Learning-Based Stocks and Flows Modeling of Road Infrastructure. *J. Ind. Ecol.* **2022**, *26* (1), 44–57. https://doi.org/10.1111/jiec.13232.

(109) Tanikawa, H.; Fishman, T.; Okuoka, K.; Sugimoto, K. The Weight of Society Over Time and Space: A Comprehensive Account of the Construction Material Stock of Japan, 1945–2010. *J. Ind. Ecol.* **2015**, *19* (5), 778–791. https://doi.org/10.1111/jiec.12284.

(110) Guo, Z.; Hu, D.; Zhang, F.; Huang, G.; Xiao, Q. An Integrated Material Metabolism Model for Stocks of Urban Road System in Beijing, China. *Sci. Total Environ.* **2014**, *470–471*, 883–894. https://doi.org/10.1016/j.scitotenv.2013.10.041.

(111) Guo, Z.; Shi, H.; Zhang, P.; Chi, Y.; Feng, A. Material Metabolism and Lifecycle Impact Assessment towards Sustainable Resource Management: A Case Study of the Highway Infrastructural System in Shandong Peninsula, China. *J. Clean. Prod.* **2017**, *153*, 195–208. https://doi.org/10.1016/j.jclepro.2017.03.194.

(112) Pauliuk, S.; Heeren, N.; Berrill, P.; Fishman, T.; Nistad, A.; Tu, Q.; Wolfram, P.; Hertwich, E. G. Global Scenarios of Resource and Emission Savings from Material Efficiency in Residential Buildings and Cars. *Nat. Commun.* **2021**, *12* (1), 5097. https://doi.org/10.1038/s41467-021-25300-4.

(113) Deetman, S.; Marinova, S.; van der Voet, E.; van Vuuren, D. P.; Edelenbosch, O.; Heijungs, R. Modelling Global Material Stocks and Flows for Residential and Service Sector Buildings towards 2050. *J. Clean. Prod.* **2020**, *245*, 118658. https://doi.org/10.1016/j.jclepro.2019.118658.
